# Plasma lipopolysaccharide-binding protein (LBP), a proxy for intestinal permeability, relates to clinicopathological and immunological disease activity in inflammatory bowel disease patients at diagnosis and during disease course

**DOI:** 10.1093/ecco-jcc/jjag146

**Published:** 2026-10-09

**Authors:** Daniëlle M H Barendregt, Bastiaan Tuk, Daniëlle H Hulleman-van Haaften, Anouk E Camman, Beatriz Calado, Meike H Grillmaier, Willem K Smits, Michail Doukas, Johanna C Escher, Lissy de Ridder, Janneke N Samsom

**Affiliations:** Laboratory of Pediatrics, Division of Gastroenterology and Nutrition, Erasmus University Medical Center-Sophia Children’s Hospital, Rotterdam, 3000 CA, The Netherlands; Laboratory of Pediatrics, Division of Gastroenterology and Nutrition, Erasmus University Medical Center-Sophia Children’s Hospital, Rotterdam, 3000 CA, The Netherlands; Laboratory of Pediatrics, Division of Gastroenterology and Nutrition, Erasmus University Medical Center-Sophia Children’s Hospital, Rotterdam, 3000 CA, The Netherlands; Department of Pediatric Gastroenterology, Erasmus University Medical Center-Sophia Children’s Hospital, Rotterdam, 3000 CA, The Netherlands; Laboratory of Pediatrics, Division of Gastroenterology and Nutrition, Erasmus University Medical Center-Sophia Children’s Hospital, Rotterdam, 3000 CA, The Netherlands; Laboratory of Pediatrics, Division of Gastroenterology and Nutrition, Erasmus University Medical Center-Sophia Children’s Hospital, Rotterdam, 3000 CA, The Netherlands; Laboratory of Pediatrics, Division of Gastroenterology and Nutrition, Erasmus University Medical Center-Sophia Children’s Hospital, Rotterdam, 3000 CA, The Netherlands; Department of Pathology, Erasmus University Medical Center, Rotterdam, 3000 CA, The Netherlands; Department of Pediatric Gastroenterology, Erasmus University Medical Center-Sophia Children’s Hospital, Rotterdam, 3000 CA, The Netherlands; Department of Pediatric Gastroenterology, Erasmus University Medical Center-Sophia Children’s Hospital, Rotterdam, 3000 CA, The Netherlands; Department of Pediatrics, Willem-Alexander Children’s Hospital, Leiden University Medical Center, Leiden, 2300 RC, The Netherlands; Laboratory of Pediatrics, Division of Gastroenterology and Nutrition, Erasmus University Medical Center-Sophia Children’s Hospital, Rotterdam, 3000 CA, The Netherlands

**Keywords:** lipopolysaccharide-binding protein, intestinal inflammation, epithelial barrier breach

## Abstract

**Background and Aims:**

Intestinal barrier healing is the treatment goal in ulcerative colitis (UC) and Crohn’s disease (CD). Despite this, systematic analyses of the relationship between intestinal permeability and clinicopathological and immunological disease activity at diagnosis and during follow-up are lacking. Therefore, we assessed this relationship using plasma lipopolysaccharide-binding protein (LBP)—an indirect biomarker of systemic exposure to bacterial endotoxins—as a proxy for intestinal permeability, in a uniquely well-characterized pediatric inflammatory bowel disease (IBD) cohort.

**Methods:**

LBP was measured in 1083 longitudinal plasma samples of newly diagnosed pediatric UC (*n* = 61), CD (*n* = 115) (PIBD-SETQuality and TISKids prospective cohorts; median follow-up 628 days), and controls (IBD-negative, *n* = 33; healthy, *n* = 28). Its relationship with clinicopathological parameters and plasma immune proteomics was examined.

**Results:**

Plasma LBP was elevated and associated with clinicopathological disease activity in therapy-naive CD and UC. In UC, but not CD, LBP related to disease extent, and strongly and independently correlated to endoscopic and histological disease activity, providing additional explanatory value beyond routinely used blood-based biomarkers and inflammatory mediators (including CRP and IL-6). LBP correlated with disease-specific immune profiles linked to severe disease (IL-17/neutrophil/IFN-γ-related profile in UC; IFN-γ-related profile in CD). LBP decreased after induction therapy with corticosteroids in UC, and after exclusive enteral nutrition or anti-TNF (but not corticosteroids) in CD. LBP remained associated with clinicopathological disease activity during follow-up, particularly in UC.

**Conclusions:**

Plasma LBP relates to clinicopathological and immunological disease activity in IBD—especially UC—at diagnosis and during follow-up, and provides complementary information to routinely used blood-based biomarkers, arguing this simple, inexpensive parameter should be (re)considered for monitoring mucosal inflammation in UC.

## Graphical abstract

**Graphical Abstract:**
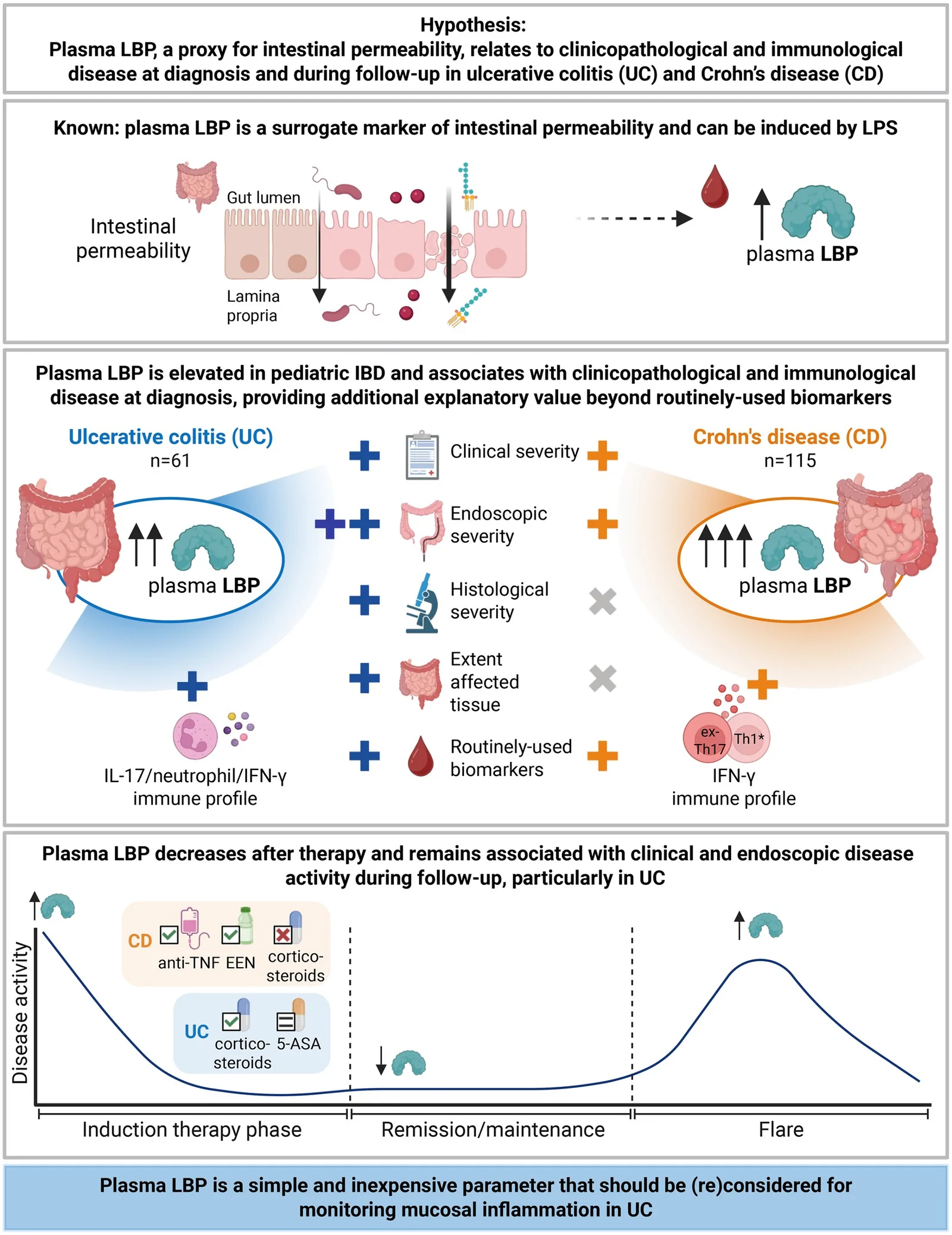

## 1. Introduction

Inflammatory bowel disease (IBD) is a chronic gastrointestinal disease, which includes ulcerative colitis (UC) and Crohn’s disease (CD). While UC is a superficial mucosal inflammation restricted to the colon, CD can affect the entire gastrointestinal tract and cause deep mucosal and transmural inflammatory lesions. Approximately 10% of IBD patients have pediatric-onset disease, which is generally associated with a more complicated disease course compared to adult-onset IBD.^1^ IBD is a multifactorial disease caused by a complex interplay of genetic predisposition, environmental factors, and gut microbiota composition.^2^ The aberrant microbial–immune interaction drives pathology. In consequence, immune suppression and immune modulation are the cornerstones of current treatment strategies.^3^

The immune responses that drive intestinal pathology in IBD are highly heterogeneous, which explains the large interindividual differences in efficacy of immune-targeting therapies.^4^ Different branches of the host immune system are involved, including neutrophils—which can quickly respond and clear invasive bacteria, but in the process cause extensive tissue damage^5^—and different types of CD4^+^ T helper (Th) cells^6^—which act in an antigen-specific manner and generate immunological memory allowing them to quickly reactivate upon re-exposure to their antigen. We have previously demonstrated that inflammatory plasma immune protein profiles and intestinal and circulating gut-homing (CD38^+^CD62L^neg^)^7^ CD4^+^ memory Th cells (T_M_) relate to immune heterogeneity and response to therapy in pediatric CD and UC.^8–10^ In particular, at time of diagnosis, pediatric CD patients who develop a severe disease course have high frequencies of circulating gut-homing pathogenic ex-Th17/Th1* T_M_ (defined by surface expression of CCR4, CXCR3, and CCR6), which differentiate from Th17 T_M_ (key producers of the cytokine IL-17A) thereby gaining the capacity to produce IFN-γ.^9^^,^^11^ In line with this, these severe CD patients have concomitantly high plasma concentrations of IFN-γ and IFN-γ-related immune proteins.^9^^,^^10^ In contrast, pediatric UC patients who develop a severe disease course have no detectible changes in subsets of circulating gut-homing T_M_ cell frequencies, but exhibit increased plasma concentrations of IL-17A-, neutrophil-, and IFN-γ-associated immune proteins, and increased infiltration of IL-17A-producing cells and neutrophils in lesional intestinal biopsies compared to IBD-negative (IBDneg) controls.^9^^,^^10^

Aberrant immune responses in IBD target intestinal microbes, including yeast and bacteria, and self-antigens.^12^ Due to epithelial barrier damage and increased intestinal permeability, the immune system of IBD patients can be exposed to microbial products, likely fueling the inflammatory response. Indeed, bacterial DNA has been detected in the blood of both UC and CD patients, with higher concentrations observed during active disease.^13–16^ This process may occur in early stages as increased intestinal permeability assessed by the lactulose/mannitol ratio test has been associated with later development of CD.^17^ Restoration of the intestinal barrier would break the cycle of microbial translocation, microbial product exposure, immune reactivation, and tissue damage. It is therefore not surprising that restoration of epithelial barrier integrity, assessed by endoscopy or histology, has become an important treatment goal in recent years.^18^ Although endoscopic mucosal healing is associated with lower relapse rates,^19^ several studies demonstrate that microscopic inflammation detected by histological assessment may persist despite endoscopic healing.^18^^,^^20^ Full resolution of intestinal inflammation is only achieved upon histological mucosal healing and restoration of the epithelial barrier. Endoscopic and histological assessments are invasive, limiting their use for routine monitoring, particularly in pediatric patients.^1^ Routinely used blood-based biomarkers, including C-reactive protein (CRP) which strongly relates to high concentrations of interleukin-6 (IL-6), are useful to detect increased systemic inflammation, but have limited sensitivity and specificity for endoscopic healing.^18^^,^^21–23^ Fecal concentrations of calprotectin (FCP), a neutrophil/macrophage-derived antimicrobial peptide, provide a valuable clinical biomarker for degree of endoscopic disease.^18^^,^^23^ However, the relationship between FCP and the severity or extent of endoscopic mucosal inflammation is not linear. At higher concentrations, FCP values no longer correlate proportionally to the degree of inflammation.^24^ Hence, biomarkers reflecting intestinal permeability and (microscopic) mucosal inflammation are desired in IBD treatment.

The soluble lipopolysaccharide (LPS)-binding protein (LBP) is an indirect biomarker of systemic exposure to bacterial endotoxins and is associated with impaired intestinal barrier function. As such, LBP is commonly used as a surrogate marker of microbial translocation.^25–27^ LBP belongs to a diverse group of acute-phase proteins with distinct kinetics, regulatory pathways, and immunological functions. As its name suggests, LBP binds bacterial endotoxins, including LPS, and transfers it to CD14, a crucial step in LPS-induced Toll-like receptor 4 (TLR4) activation.^28^^,^^29^ Research in sepsis patients, who exhibit high plasma LBP concentrations, demonstrated that while at lower concentrations LBP facilitates LPS-induced TLR4 activation, at higher concentrations LBP inhibits innate immune cell activation.^30^ LBP is primarily produced by hepatocytes upon stimulation with LPS or the cytokines IL-6, IL-1β, TNF-α, and the glucocorticoid dexamethasone.^31–33^ LBP has been reported to be elevated in both UC and CD patients,^34–38^ and to associate with clinical disease activity in both UC and CD and endoscopic disease activity in CD.^36–38^ However, this relationship with disease activity has not been observed in all cohorts.^34^ In addition, there are contradictory reports on the association between LBP and disease characteristics, including disease extent in UC and complicated disease behavior in CD.^34^^,^^37^^,^^38^ This raises the question of whether plasma LBP concentrations accurately reflect endoscopic and histological disease activity, associate with disease characteristics, and/or relate to the detrimental immune response in individual IBD patients. Moreover, it remains unknown whether LBP provides complementary biological information on clinicopathological disease activity beyond routinely used biomarkers and other acute phase proteins, such as CRP and IL-6.

Therefore, we performed an in-depth investigation of the relationship between plasma LBP concentrations and clinicopathological parameters, treatment response, disease course, and immune profiles in UC and CD at diagnosis and throughout follow-up, using a uniquely well-characterized pediatric IBD cohort.

## 2. Material and methods

### 2.1. Patients

At diagnosis, therapy-naive pediatric IBD patients were prospectively enrolled into the TISKids randomized controlled trial (RCT),^39^ LONGITUDINAL inception cohort, or PIBD-SETQuality inception cohort^40^ at Erasmus Medical Center, Rotterdam, The Netherlands. Patients were classified as CD (*n* = 115), UC (*n* = 57), or IBD-Unclassified (*n* = 4; grouped with UC). Patients with gastrointestinal complaints but negative IBD diagnoses after endoscopy and histological biopsy evaluation by the pathologist were included as IBD negative controls (IBDneg, *n* = 33). In collaboration with the orthopedic and anesthesiology departments, blood from pediatric healthy controls (HC; *n* = 28; without intestinal complaints) was collected. All studies were approved by Erasmus Medical Center institutional ethical review board (MEC-2015-080, MEC-2007-335, MEC-2016-321). Informed consent was obtained from patients and parents according to Dutch regulations.

### 2.2. Sample and clinical data collection

Blood samples (*n* = 1083) were collected at diagnosis and at scheduled and unscheduled follow-up visits. Whole blood was used for flow cytometric analysis, and to measure biochemical markers including hemoglobin, erythrocyte sedimentation rate (ESR), CRP, and albumin. EDTA plasma was collected, stored at −80 °C and used to measure the concentration of LBP and 92 immune proteins. Intestinal biopsies were obtained via endoscopy at time of diagnosis–and in some cases also at follow-up visits–from macroscopically non-affected and macroscopically affected ileum and colon, and used for immunohistochemistry and histological scoring (including granuloma detection) by an experienced gastrointestinal pathologist. Stool samples for measuring FCP were only collected as per the physician’s discretion and not part of the standard study protocol. Simplified Endoscopic Activity Score for Crohn’s Disease (SES-CD) and Ulcerative Colitis Endoscopic Index of Severity (UCEIS) scores were assessed during endoscopy at diagnosis and during follow-up. Complete SES-CD scores could not be obtained for 22 CD patients at time of diagnosis, because the ileum was not reached. UCEIS sub-scores of all colonic segments were summed to the total UCEIS score (range 0-40). In CD patients, location and behavior of disease were classified following the Paris classification.^41^ For UC patients, extent of disease was also classified using the Paris classification. Clinical disease activity was assessed for CD using the weighted pediatric CD Activity Index (wPCDAI)^42^ and for UC using the pediatric UC Activity Index (PUCAI).^43^

Median follow-up time of the pediatric IBD patients was 628 days from diagnosis. Per patient, samples were collected at scheduled visits: visit 0 (baseline) until visit 11 (approximately 7 years post-diagnosis). As TISKids was an RCT, follow-up visits were tightly scheduled at 10-14 weeks (visit 2) and 52 weeks (visit 4) after start of induction therapy. As PIBD-SETQ was an observational inception cohort, the time from start of induction therapy to the follow-up visit varied between patients; an overview of the timing of scheduled PIBD-SETQ visits relative to therapy initiation can be found in Table S1. Unscheduled visits (*n* = 76) were conducted for various reasons, including follow-up endoscopy (part of the TISKids RCT protocol), hospitalization, or start of biological therapy.

### 2.3. LPS-binding protein (LBP) ELISA

LBP concentrations were measured in EDTA plasma (1000× diluted) using the Human LBP DuoSet ELISA (R&D systems, cat. DY870-05) according to the manufacturer’s instructions.

### 2.4. Plasma immune protein measurement using proximity extension assay

Concentrations of 92 immune proteins were determined in EDTA plasma samples using a proximity extension assay (92 Inflammation Panel, Olink, Sweden), as described previously.^10^ Concentrations are expressed as normalized protein expression (NPX; log_2_, arbitrary units) after normalization of qPCR values. Fourteen proteins were excluded from downstream analysis because >50% of samples were below the limit of detection.

### 2.5. Immunohistochemistry and histological scoring

Tissue biopsies were formalin-fixed and paraffin-embedded. Sections (4 µm thick) were cut and mounted on polysine adhesion glass slides (Thermo Fisher Scientific, Waltham, MA, USA), deparaffinized in xylene, and rehydrated in ethanol. For H&E staining, sections were stained for 4 min with Harris’ hematoxylin and subsequently for 20 s with eosin (yellow, alcoholic) using a Leica Multistainer ST 5020. For immunohistochemistry staining, endogenous peroxidase activity was quenched with 3% H_2_O_2_ in PBS for 20 min followed by antigen retrieval by microwave treatment in citrate buffer (10 mM, pH 6.0). Sections were blocked in 10% normal human serum (AB serum, Sanquin, The Netherlands) plus 10% normal rabbit or horse serum diluted in buffer (10 mM Tris, 5 mM EDTA, 0.15 M NaCl, 0.25% gelatin, 0.05% Tween-20, pH 8) for 1 h. Antibodies and isotype controls were incubated overnight at 4 °C. Slides were incubated with biotinylated secondary antibodies for 1 h at room temperature. Biotinylated antibodies were detected using a biotin–avidin complex (Vectastain ABC Elite Kit, Vector Laboratories) and 3,3′-diaminobenzidine tetrahydrochloride (Sigma-Aldrich). Nuclei were counterstained with hematoxylin (Vector Laboratories). Subsequently, sections were dehydrated, immersed in xylene, and mounted in Entallan (Sigma-Aldrich). Stained slides were scanned and visualized using a 3DHistech PANNORAMIC® MIDI II equipped with a 20× lens, 68 PANNORAMIC® digital scanner software 3.0.7, and SlideViewer 2.5. See the Table S2 for more details.

H&E-stained biopsies were scored by an experienced gastrointestinal pathologist using a modification of the Global Histological Activity Score (GHAS).^44^^,^^45^ In the modified GHAS, the parameters “presence of granuloma” and “number of affected biopsies” were excluded because the cohort included both CD and UC patients and maximally two biopsies were taken from the same anatomic region. Per patient, the highest GHAS scores in colonic and ileal biopsies were selected as the colonic and ileal GHAS scores for analysis. Presence of granuloma was considered a separate parameter.

Immunohistochemistry staining of IL17A was scored semi-quantitatively by two independent observers, as published before.^46^ Numbers of calprotectin-positive cells were counted (maximum 200 positive cells/mm^2^). Per patient, the highest score of each immunohistochemistry parameter in colonic and in ileal biopsies was used for data visualization and statistical analyses.

### 2.6. Statistics

After checking for a normal distribution using the Shapiro–Wilk test, *P*-values were calculated using the appropriate parametric or non-parametric test and corrected for multiple testing when needed. Statistical tests are specified in the figure legends. *P*-values <.05 were considered significant.

To assess whether LBP was independently associated with clinical, endoscopic, and histological disease activity scores after correction for other biomarkers, we performed multivariable linear regression analyses (R package stats). To assess whether LBP was of incremental value beyond routinely measured biomarkers when assessing clinicopathological scores, we fitted multivariable linear regression models with and without LBP and tested whether LBP improved model fit using F tests. The relationship between LBP concentrations and clinical disease activity scores over time was assessed using a linear mixed model considering time as a co-variate and patient as a random effect (R package lme4). Model coefficients were estimated using restricted maximum likelihood. Statistical significance of fixed effects was assessed using Type II Wald χ² tests (R package car). Analyses and data visualization were performed in R version 4.5.2^47^ and GraphPad Prism version 9 (La Jolla, CA, USA).

## 3. Results

### 3.1. At time of diagnosis, plasma LBP concentrations are increased in both UC and CD, highest in CD, and correlate with clinical disease activity scores

To determine whether plasma LBP concentrations are increased in IBD patients at time of diagnosis, we measured LBP in plasma of therapy-naive pediatric UC (*n* = 61; including four IBD-Unclassified patients) and CD (*n* = 115) patients, pediatric IBD-negative controls with intestinal complaints (IBDneg; *n* = 33), and age-matched pediatric healthy controls (pHC; *n* = 28) (Table 1). Both newly diagnosed UC and CD patients had significantly increased plasma LBP concentrations compared to controls (Figure 1A). IBDneg controls and pHC had comparably low LBP concentrations. Strikingly, despite the fact that CD has skip lesions while UC affects a more continuous surface area, LBP concentrations were higher in CD than in UC, with some CD patients reaching an LBP plasma concentration of over 30 000 ng/mL (Figure 1A). In both UC and CD patients, LBP concentrations positively correlated with clinical disease activity scores (PUCAI in UC; wPCDAI in CD) at time of diagnosis (Figure 1B) with comparable *R* (UC *R* = 0.4; CD *R* = 0.45). In both UC and CD, LBP did not associate with age or sex (Figure S1A, B).

**Figure 1.**
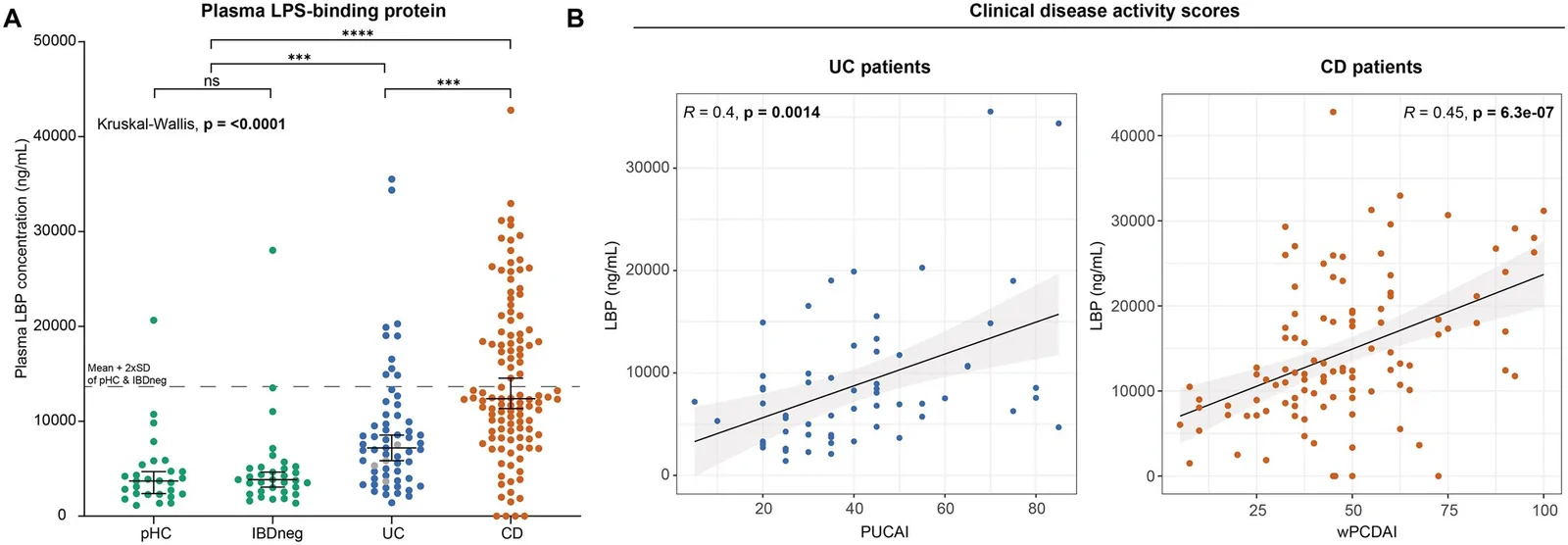
Plasma LBP concentrations are increased in pediatric IBD patients and associate with clinical disease activity scores at time of diagnosis. (A) LBP concentrations in plasma of pediatric healthy controls (pHC), IBD-negative controls with gastrointestinal complaints (IBDneg), and ulcerative colitis (UC) patients and Crohn’s disease (CD) patients at time of diagnosis. Gray dots depict IBD-unclassified patients. Dotted line indicates the mean + 2SD of the grouped controls (pHC and IBDneg). Lines and error bars depict median with 95% confidence interval. Kruskal–Wallis test followed by Dunn’s tests corrected for multiple comparisons. (B) Correlation of LBP concentrations to the clinical disease activity scores Pediatric Ulcerative Colitis Activity Index (PUCAI) and weighted Pediatric Crohn’s Disease Activity Index (wPCDAI) in UC and CD patients, respectively. Spearman’s correlation. ns, not significant, *P < .05, **P < .01, ***P < .001, ****P < .0001.

**Table 1.** Cohort characteristics at time of diagnosis.

	Crohn’s disease, *N* = 115	Ulcerative colitis, *N* = 57	IBD-Unclassified, *N* = 4	IBD Negative, *N* = 33	Healthy controls, *N* = 28	*P*-value
**Age**						**.030**
** Mean (SD)**	13.92 (2.78)	14.61 (2.18)	15.58 (1.44)	13.60 (2.99)	12.51 (3.19)	
** Median (Q1, Q3)**	14.47 (12.12, 16.22)	15.08 (12.75, 16.44)	15.67 (14.53, 16.62)	14.52 (11.35, 15.59)	12.11 (10.09, 15.14)	
** Min, max**	6.19, 17.91	9.60, 17.81	13.77, 17.21	6.28, 17.71	6.92, 18.80	
**Female sex**	56 (49%)	33 (58%)	2 (50%)	18 (55%)	14 (50%)	.8
**CD characteristics**						
**wPCDAI**						
** Mean (SD)**	48 (21)					
** Median (Q1, Q3)**	45 (35, 60)					
** Min, max**	5, 100					
** Unknown**	5					
**SES-CD**						
** Mean (SD)**	13 (7)					
** Median (Q1, Q3)**	10 (7, 17)					
** Min, max**	2, 31					
** Unknown**	22					
**Disease location**						
** L1**	43 (40%)					
** L2**	13 (12%)					
** L3**	52 (48%)					
** Unknown**	7					
**Disease behavior**						
** B1**	92 (85%)					
** B2**	10 (9.3%)					
** B3**	4 (3.7%)					
** B2B3**	2 (1.9%)					
** Unknown**	7					
**Perianal disease**						
** No**	95 (88%)					
** Yes**	13 (12%)					
** Unknown**	7					
**UC characteristics**						
**PUCAI**						
** Mean (SD)**		41 (19)	36 (23)			
** Median (Q1, Q3)**		38 (25, 50)	38 (18, 55)			
** Min, max**		5, 85	10, 60			
** Unknown**		1	0			
**UCEIS**						
** Mean (SD)**		15 (8)	13 (7)			
** Median (Q1, Q3)**		15 (9, 21)	13 (9, 18)			
** Min, max**		2, 33	5, 21			
** Unknown**		5	0			
**Disease extent**						
** E1**		6 (11%)	1 (25%)			
** E2**		10 (18%)	0 (0%)			
** E3**		6 (11%)	0 (0%)			
** E4**		34 (61%)	3 (75%)			
** Unknown**		1	0			
**Fecal calprotectin (µg/g)**						
** Mean (SD)**	1915 (1895)	3231 (4109)	1241 (968)			.080
** Median (Q1, Q3)**	1100 (620, 2520)	1732 (1004, 4019)	845 (533, 2344)			
** Min, max**	15, 9727	89, 19609	533, 2344			
** Unknown**	6	10	1			
**Fecal calprotectin >250 µg/g**	106 (97%)	45 (96%)	3 (100%)			
** Unknown**	6	10	1			
**CRP (mg/L)**						
** Mean (SD)**	32 (35)	10 (18)	12 (23)			**<.001**
** Median (Q1, Q3)**	20 (7, 44)	2 (1, 11)	1 (0, 24)			
** Min, max**	0, 173	0, 113	0, 46			
** Unknown**	3	0	0			
**ESR (mm/h)**						
** Mean (SD)**	34 (20)	28 (22)	44 (36)			.078
** Median (Q1, Q3)**	30 (20, 47)	24 (15, 38)	42 (19, 69)			
** Min, max**	3, 83	2, 111	2, 90			
** Unknown**	8	4	0			
**Albumin (g/L)**						
** Mean (SD)**	36 (6)	39 (6)	38 (7)			**.005**
** Median (Q1, Q3)**	36 (32, 40)	39 (36, 43)	39 (32, 44)			
** Min, max**	21, 49	23, 49	29, 45			
** Unknown**	10	1	0			
**Hemoglobin (mmol/L)**						
** Mean (SD)**	7.06 (0.97)	6.95 (1.29)	7.33 (1.54)			.9
** Median (Q1, Q3)**	7.10 (6.50, 7.80)	7.20 (6.20, 7.90)	7.45 (6.15, 8.50)			
** Min, max**	4.50, 9.20	3.40, 9.20	5.40, 9.00			
** Unknown**	9	0	0			

Significant *P*-values (<.05) are reported in bold.

### 3.2. At diagnosis, plasma LBP associates with both endoscopic scores and histological scores and disease extent in UC; it does not associate with histological scores, complicated disease, disease location, or granuloma in CD

To assess whether plasma LBP associates with intestinal tissue destruction and immune infiltration, we determined its relationship to endoscopic scores (UCEIS in UC; SES-CD in CD) at diagnosis. As anticipated, plasma LBP significantly related to endoscopic disease activity, but the correlation was much stronger in UC (total UCEIS *R* = 0.71; highest UCEIS sub-score *R* = 0.55) than in CD (SES-CD *R* = 0.28) (Figures 2A, B and S2A). The strong association with both the total UCEIS score and highest UCEIS sub-score indicates that in UC, LBP associates with the overall extent of disease as well as with the severity of inflammation and tissue damage within the most affected intestinal segment. In agreement, therapy-naive UC patients with extensive disease and pancolitis had higher LBP concentrations compared to patients with proctitis and left-sided colitis (Figure 2C). Moreover, plasma LBP concentrations significantly correlated with the histological disease activity (scored using the modified GHAS^44^^,^^45^) of the most affected colonic biopsy in each UC patient at time of diagnosis (Figure 2E). In contrast, in CD patients, LBP did not associate with disease location, perianal disease, or the presence of granulomas at time of diagnosis (Figure 2D). A non-significant increase in LBP was observed in patients with penetrating and/or stricturing disease behavior (B2/B3), though the analysis was limited by few patients with B2/B3 disease. To assess whether LBP was related to the extent of affected intestinal surface in CD, similar to UC, we correlated LBP to the SES-CD sub-score “extent of affected surface,” but this did not yield a significant correlation (Figure S2B), suggesting intestinal permeability in CD is not linearly related to size of the affected tissue as in UC. In addition, LBP did not relate to the histological disease activity scores in the most affected ileal and colonic biopsy of CD patients (Figures 2F and S2C). The lack of relationship with histological disease scores may reflect the “patchy” nature of CD, which makes biopsy sampling challenging. Moreover, the depth of lesions in CD may be of greater importance to intestinal permeability and LBP concentrations, but is not captured in the histological GHAS score. The slightly increased LBP concentrations in patients with penetrating and/or stricturing disease would agree with this hypothesis. Furthermore, stratification by disease location revealed no differences in the correlation of LBP with SES-CD and GHAS, though analyses were limited by few patients with isolated colonic CD (Figures 2F and S2A).

**Figure 2.**
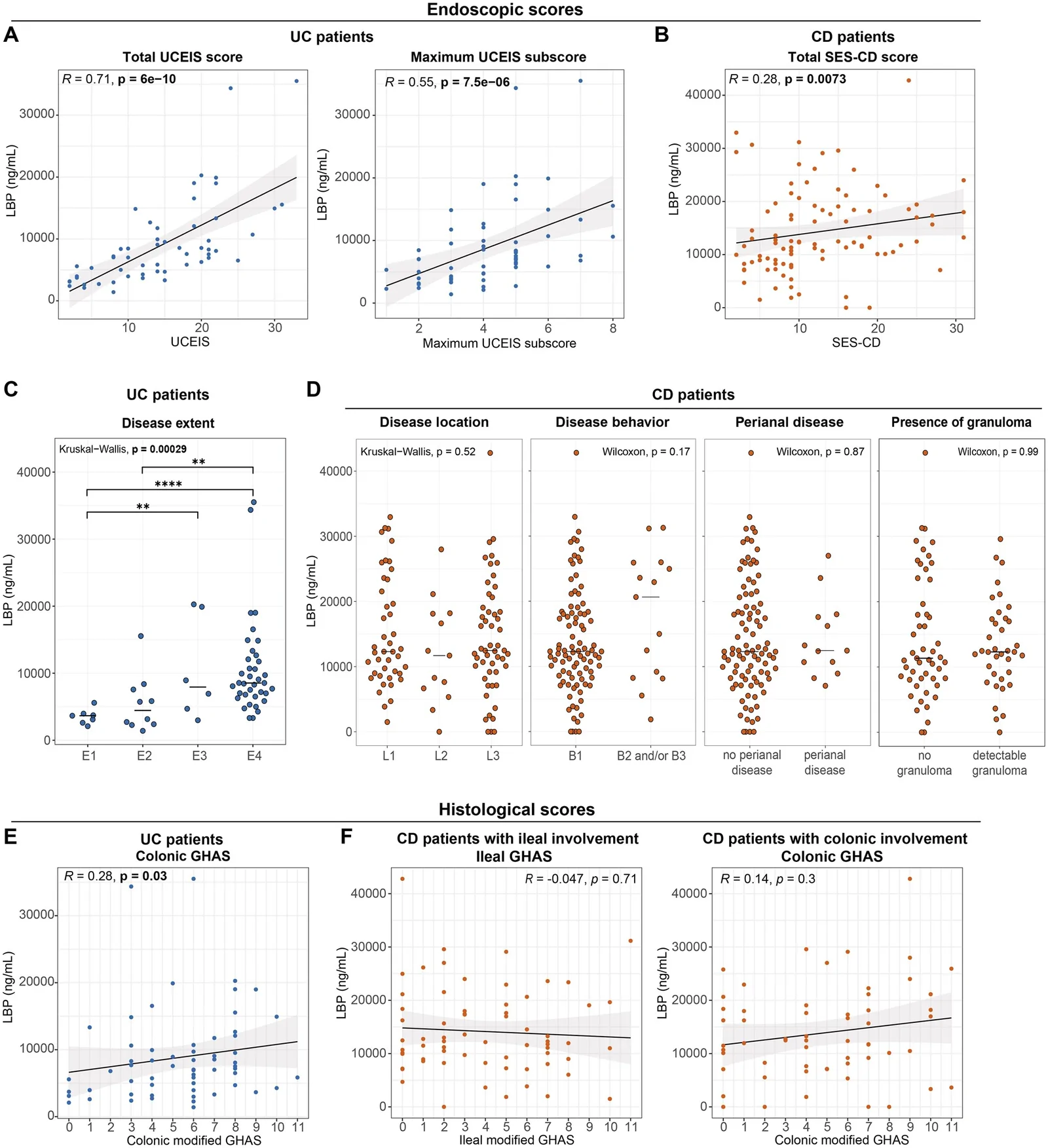
LBP correlates with endoscopic and histological disease activity scores at diagnosis and associates with disease extent in UC but not with complicated disease in CD. (A, B) Correlation of LBP concentrations to endoscopic scores UCEIS (both the total and the maximum sub-score) (A) and SES-CD (B) in UC and CD patients, respectively. Spearman’s correlation. (C) LBP concentrations in UC patients with increasing disease extent, according to the Paris classification. E1 = proctitis, E2 = left-sided colitis, E3 = extensive colitis, E4 = pancolitis. Kruskal–Wallis and Wilcoxon test. (D) LBP concentrations in CD patients per disease location, with or without complicated disease behavior, with or without perianal disease according to the Paris classification, and with or without granulomas (from left to right). L1 = ileal, L2 = colonic, L3 = ileocolonic. B1 = inflammatory disease behavior, B2 and/or B3 = stricturing and/or penetrating disease behavior. Kruskal–Wallis and Wilcoxon tests. (E) Correlation of LBP concentrations to the modified global histological activity scores (GHAS) in the most affected colonic biopsy of UC patients. Spearman’s correlations. (F) Correlation of LBP concentrations to the modified global histological activity scores (GHAS) in the most affected ileal and colonic biopsies of CD patients with macroscopic ileal and colonic involvement, respectively. Macroscopic ileal and colonic involvement was based on the disease location according to the Paris classification. Spearman’s correlations. ns, not significant, *P < .05, **P < .01, ***P < .001, ****P < .0001.

Together, these data demonstrate that, at time of diagnosis, prior to therapy, plasma LBP concentrations are increased in both pediatric UC and CD, with a greater increase observed in CD. In UC, plasma LBP is linearly related to the size of affected tissue surface as well as endoscopic and histological severity of intestinal disease. In contrast, in CD, LBP does not relate to the affected tissue surface and histological disease activity, and is only weakly associated with endoscopic disease activity, suggesting high plasma LBP concentrations in CD may relate to other disease processes, such as depth of the inflammatory lesion or the translocation of specific bacteria.

### 3.3. At diagnosis, plasma LBP correlates with routinely used blood-based biomarkers, but not FCP

To assess the relationship between plasma LBP and blood-based and fecal biochemical biomarkers routinely used for monitoring IBD disease activity, we related plasma concentrations of LBP to albumin, CRP, ESR, hemoglobin (Hb), and FCP. When comparing UC and CD patients at diagnosis, we found significant differences in CRP—in line with previous studies^48–51^—and albumin concentrations (Table 1**)**. Differences in FCP and ESR were not statistically significant. In both pediatric UC and CD patients, plasma LBP positively correlated with CRP and ESR, but not FCP, at time of diagnosis (Figure 3A). The lack of correlation with FCP may be due to the fact that only very few patients had an FCP of <250 µg/g at diagnosis (Table 1), and could be indirectly caused by the non-linear relationship between high FCP concentrations and severity or extent of mucosal inflammation.^18^^,^^24^ In accordance with this, decreased FCP concentrations at later timepoints after induction therapy did correlate with LBP concentrations in CD (see Figure 5E, 6C and S6A). Furthermore, LBP negatively correlated with Hb and albumin in both CD and UC patients. As increased intestinal permeability in IBD can cause loss of albumin via the gastrointestinal tract,^52^ this agrees with the link between plasma LBP and intestinal barrier disruption.

**Figure 3.**
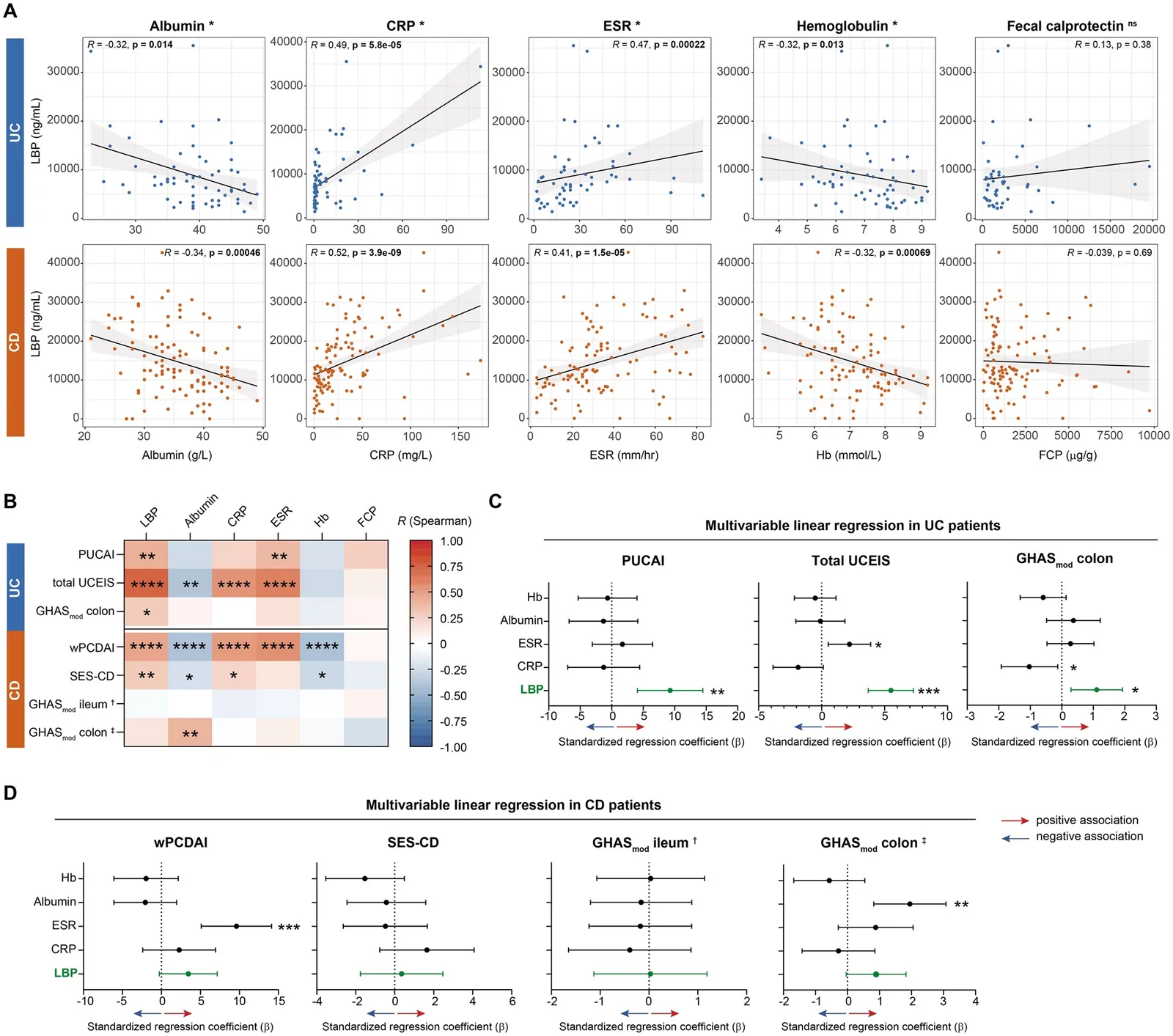
LBP associates with blood-based biochemical parameters and clinical, endoscopic, and histological disease activity, but not fecal calprotectin, at time of diagnosis. (A) Correlations of LBP concentrations vs albumin, CRP, ESR, hemoglobin (Hb) and fecal calprotectin (FCP) in UC (top) and CD (bottom). Spearman’s correlations. *P < .05; ns *P* > .05. (B) Correlation heatmap of fecal and blood-based biomarkers vs clinical disease, and endoscopic and histological activity scores in UC and CD patients at diagnosis. Color represents the R of Spearman’s correlations. Stars represent raw *P*-values. For correlation with histological activity in CD, we selected patients with macroscopic ileal involvement (†) and colonic involvement (‡), based on the disease location established according to the Paris classification. (C, D) Forest plots depicting the standardized regression coefficients (β) and 95% confidence intervals (CI) of multivariable linear regression models including LBP, CRP, ESR, albumin, and Hb as covariates and clinical, endoscopic, and histological disease scores as dependent variables in UC (C) and CD (D). Covariates were standardized to z-scores prior to analysis to enable comparison of effect sizes across covariates. Positive coefficients indicate a positive association with the outcome, whereas negative coefficients indicate an inverse association. † in selection of patients with macroscopic ileal involvement, ‡ in selection of patients with macroscopic colonic involvement, based on the disease location established according to the Paris classification. *P < .05, **P < .01, ***P < .001, ****P < .0001.

### 3.4. In UC, plasma LBP at diagnosis is strongly and independently associated with clinical, endoscopic, and histological disease activity, and provides additional explanatory value beyond routinely used blood-based biomarkers

LBP is an acute-phase protein, like the routinely used biomarker CRP and the LBP- and CRP-inducing cytokine IL-6. Acute-phase proteins comprise a diverse group of plasma proteins with distinct kinetics, regulatory pathways, and immunological functions. Therefore, we asked whether LBP captures additional biological information associated with intestinal inflammatory activity beyond the established and routinely used blood-based biomarkers, such as CRP. To this end, we first compared its univariate correlations with clinical, endoscopic, and histological scores to those of the routinely used biomarkers. In UC, LBP showed a stronger correlation with endoscopic (UCEIS) and clinical (PUCAI) disease activity scores than the other biomarkers, and was the only biomarker significantly correlated to histological disease activity (colonic GHAS; Figure 3B). In CD, LBP correlated with clinical disease activity (wPCDAI) similarly to the other biomarkers, but more strongly with endoscopic disease activity (SES-CD, Figure 3B). FCP did not significantly correlate with clinical and endoscopic scores in either UC or CD in this cohort, while it has previously been reported to be a sensitive marker for mucosal inflammation in IBD.^18^^,^^53^^,^^54^ Again, this discrepancy may be explained by the non-linear relationship of high FCP with disease activity and the high FCP values in this therapy-naive cohort (Table 1).

To assess whether the associations of LBP with clinical, endoscopic, and histological disease activity are independent of the routinely used blood-based biomarkers, we performed multivariable linear regression analyses, including LBP and the routinely used biomarkers CRP, ESR, Hb, and albumin as predictor covariates. Both standardized (Figure 3C, D) and non-standardized (Figure S3A, B) predictor covariates were used. Standardization removes differences arising from the measurement scales of the covariates (ie, the blood-based biomarkers), allowing direct comparison of the relative strength of the independent associations of each biomarker with the clinicopathological measure (Figure 3C, D). In contrast, models using non-standardized covariates provide information on the absolute effect sizes associated with a one-unit increase in each biomarker (Figure S3A, B). In UC, plasma LBP was independently associated with higher clinical (PUCAI, *P* = .001), endoscopic (UCEIS, *P* < .001) and histological (colonic GHAS, *P* = .010) disease activity scores (Figure 3C). Each 1 µg/mL increase in LBP corresponded to increases of 1.37 points in PUCAI, 0.81 points in UCEIS, and 0.16 points in GHAS (Figure S3A, B). Comparison of the standardized regression coefficients demonstrated that in UC, plasma LBP consistently had the largest effect size across models for clinical, endoscopic, and histological disease activity (Figure 3C), indicating that LBP had the strongest independent association with the clinicopathological disease activity scores. In CD, plasma LBP was not significantly associated with clinical (wPCDAI, *P* = .07), endoscopic (SES-CD, *P* = .74), or histological (colonic GHAS in patients with macroscopic colonic involvement, *P* = .07; ileal GHAS in patients with macroscopic ileal involvement, *P* = .77) disease activity, after adjustment for the other biomarkers (Figure 3D). Together, these results demonstrate that at time of diagnosis, plasma LBP is both independently and more strongly associated with clinical, endoscopic, and histological disease activity than routinely used blood-based biomarkers in UC, whereas such independent and strong associations were not observed in CD.

To assess the additive value of plasma LBP beyond the routinely used blood-based biomarkers, we evaluated whether addition of LBP as predictor to the multivariable linear regression models (that also include the routinely used blood-based biomarkers as predictors) significantly improved model fit using F-tests. In UC, inclusion of LBP significantly improved model fit for clinical (PUCAI, Δ*R*^2^ = 0.17, *P* = .001), endoscopic (UCEIS, Δ*R*^2^ = 0.35, *P* < .0001), and histological (colonic GHAS, Δ*R*^2^ = 0.11, *P* = .01) disease activity at diagnosis, indicating that LBP provided information about the clinicopathological measures beyond that contained in the routinely used blood-based biomarkers and improved the models’ estimation of the clinicopathological scores. In contrast, addition of LBP did not significantly improve model fit for clinicopathological scores in CD. Hence, LBP provides incremental explanatory value beyond the routinely used blood-based biomarkers in UC, but not in CD.

Lastly, to assess whether LBP captures additional biological information on top of IL-6, which is an important upstream regulator of the acute-phase response, we performed additional multivariable linear regression analyses including LBP and IL-6 as predictors. In these analyses, LBP retained an independent association with clinicopathological measures after adjustment for IL-6. Specifically, LBP remained independently associated with PUCAI (*P* = .0327) and UCEIS (*P* = .000214) in UC and with wPCDAI (*P* = .000242) in CD (Figure S3C, D). In contrast, IL-6 did not retain an independent association with PUCAI, UCEIS, or wPCDAI in these models. These data suggest that although IL-6 is an important upstream regulator of the acute-phase response and inducer of LBP, LBP captures additional biological information associated with intestinal inflammatory activity in UC that is not fully reflected by IL-6 alone.

### 3.5. Plasma LBP concentrations associate with disease-specific immune responses in therapy-naive UC and CD patients

We have previously demonstrated that inflammatory plasma immune protein profiles relate to immune heterogeneity and treatment response in pediatric IBD.^8–10^ In particular, UC patients at diagnosis had increased concentrations of plasma immune proteins compared to IBDneg and pHC controls. Most pronounced were the changes in IL-17/neutrophil/IFN-γ-related proteins, like IL-17A and IL-17C, the neutrophil-associated proteins CCL3, CCL23, and OSM, and the IFN-γ-associated proteins CXCL9 and CXCL10 (Figure 4A). Our previous studies demonstrated that patients with high concentrations of these cytokines developed a more severe disease course than the other UC patients, and needed earlier escalation to anti-TNF treatment.^9^^,^^10^

**Figure 4.**
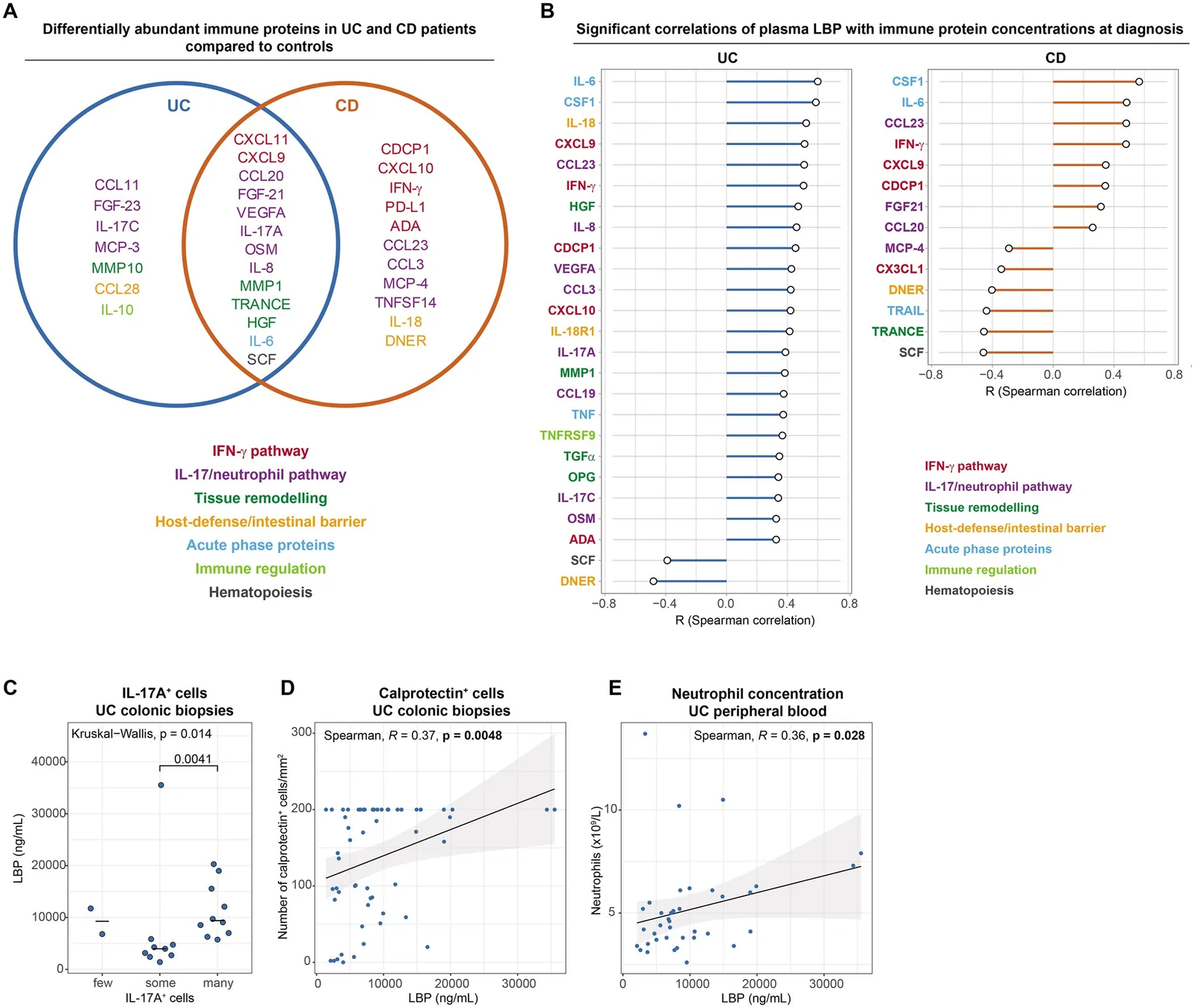
LBP associates with systemic and intestinal immune responses in IBD patients at time of diagnosis. (A) Venn diagram depicting the plasma immune proteins that were significantly differentially abundant in UC vs pHC and IBDneg controls (blue circle) or in CD vs pHC and IBDneg controls (orange circle) at time of diagnosis. Colored protein names indicate the related pathways. Student *t* tests were performed per protein across pairs of disease groups, followed by Benjamini–Hochberg correction. (B) Lollipop plots representing the significant correlations (*R*) between LBP and immune protein concentrations in plasma. Spearman’s correlations followed by Benjamin–Hochberg correction. Samples size: CD *n* = 115 and UC *n* = 58. Non-significant correlations can be found in Figure S4. (C) Semi-quantitative immunohistochemical scoring of IL-17A-producing cells in colonic biopsies of UC patients. Kruskal–Wallis and Wilcoxon tests. (D) Number of calprotectin-positive cell/mm^2^ in colonic biopsies of UC patients assessed with immunohistochemistry. Spearman’s correlation. (E) Concentration of neutrophils in peripheral blood. Spearman’s correlation.

To investigate how LBP relates to the inflammatory IL-17/neutrophil/IFN-γ-related immune profile in UC patients at diagnosis, we analyzed associations between plasma LBP concentrations and (1) 78 plasma immune proteins and (2) immune cell infiltration in intestinal biopsies assessed by immunohistochemistry. Plasma LBP was strongly associated with the inflammatory signature in UC, as it significantly correlated with IL-17/neutrophil-related proteins, including IL-8 (CXCL8), CCL3, CCL23, OSM, IL-17A, and IL17C, as well as IFN-γ-related proteins IFN-γ, CXCL9, CXCL10, and CDCP1 (Figure 4B). Consistent with these findings in plasma, higher LBP concentrations were associated with increased infiltration of IL-17A^+^ cells and calprotectin^+^ inflammatory cells in lesional colonic tissue of UC patients (Figure 4C, D). Furthermore, LBP correlated with circulating neutrophil frequencies in UC patients (Figure 4E). Together, these data indicate that in UC, high LBP concentrations are associated with an IL-17/neutrophil/IFN-γ-related immune profile, which was previously associated with more severe disease, as they have increased concentrations of IL-17/neutrophil/IFN-γ-related proteins in plasma, as well as increased infiltration of IL-17A- and calprotectin-producing cells in intestinal lesions.

In CD patients at time of diagnosis, we previously demonstrated that increased frequencies of gut-homing ex-Th17/Th1* T_M_ cells and an associated IFN-γ-related plasma protein signature identify patients with a more severe disease course^9^^,^^10^ (Figure 4A). Plasma LBP was associated with this IFN-γ-related plasma immune protein signature, as it correlated with IFN-γ, the IFN-γ-induced chemokine CXCL9 (Figure 4B). These findings suggest that elevated LBP is associated with distinct disease-specific inflammatory immune profiles in both UC and CD.

In addition to the disease-specific immune profiles, LBP strongly correlated with the acute phase protein IL-6, a key inducer of LBP, in both UC and CD, as expected^31–33^ (Figure 4B). Furthermore, in both IBD types, the positive correlation of LBP with CSF1—important for intestinal epithelial barrier function through its effect on (non-inflammatory) monocytes and macrophages^55–58^—and the negative correlation with DNER—an activator of the Notch-1 signaling pathway, important for epithelial barrier integrity^59^—may reflect an attempt of the local immune system to promote host-defense, while preventing excessive inflammation (Figure 4B). Together, these data argue that at diagnosis, prior to therapy, high LBP is not only associated with proteins that play a role in intestinal epithelial barrier function, but also with immune pathways related to therapy resistance and a severe disease course in UC and CD.^9^^,^^10^

### 3.6. Plasma LBP concentrations decrease after induction therapy with corticosteroids in UC, and after EEN and upfront anti-TNF therapy, but not corticosteroids, in CD

To investigate the relationship between treatment effectiveness and LBP, we assessed whether LBP concentrations decline after induction therapy aimed at inducing mucosal healing. As anticipated, we see a strong decrease in LBP concentrations in the first months after diagnosis in both UC and CD patients; plasma LBP concentrations were significantly lower after approximately 3 months of induction therapy compared to time of diagnosis in paired samples (Figure 5A, C). Importantly, 14 out of 90 CD (but no UC) patients still exhibited elevated LBP plasma concentrations (above the mean + 2SD of the IBDneg and pHC grouped together; 13 626.42 ng/mL) after the induction therapy phase, questioning whether remnant intestinal inflammation is present in these patients.

**Figure 5.**
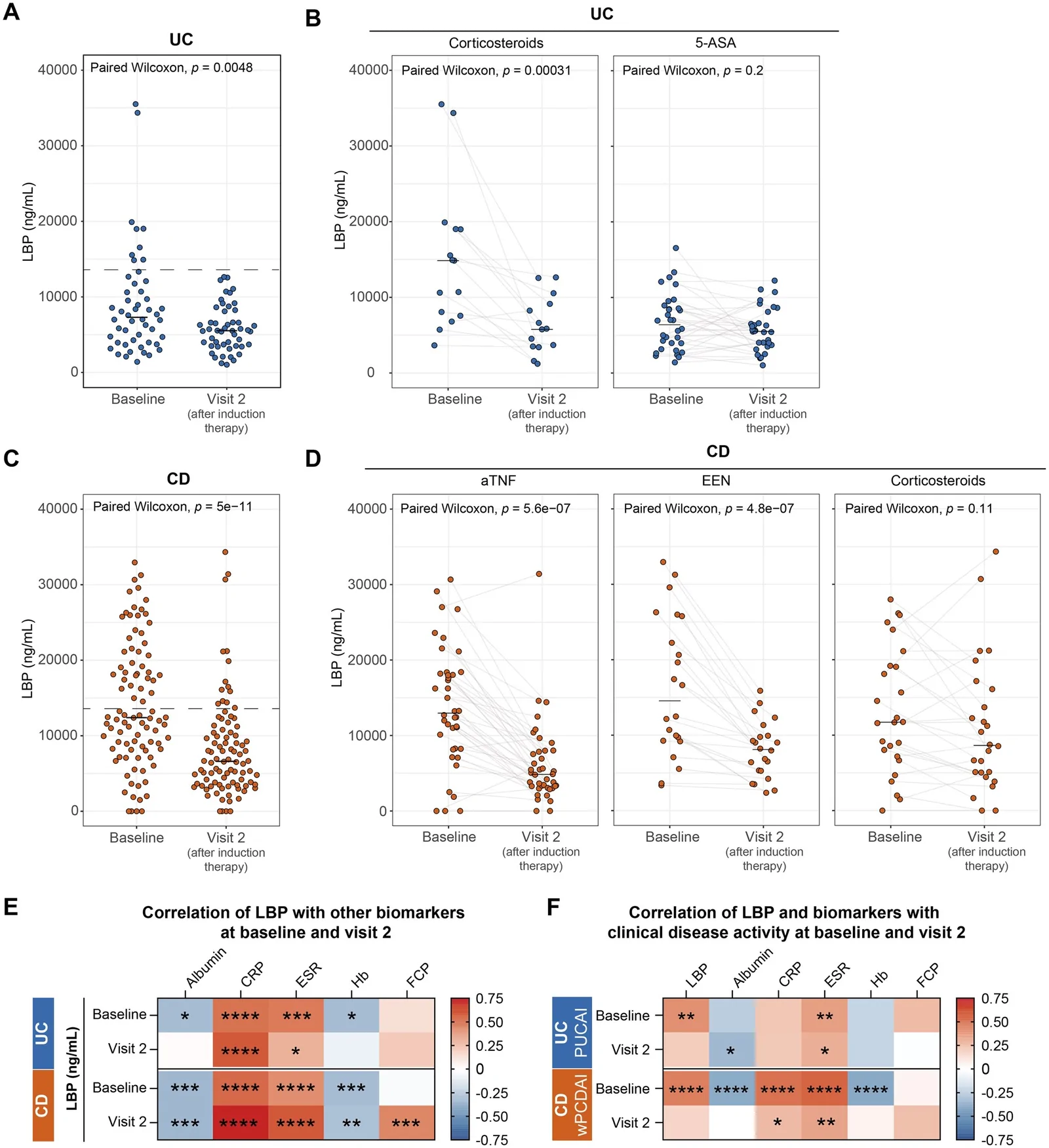
LBP concentrations are reduced by some but not all types of induction therapy, and this reduction associates with changes in immune plasma protein concentrations. (A) LBP concentrations in UC patients at time of diagnosis (baseline) and at visit 2 (∼3 months after the start of induction therapy). Dashed line indicates the mean + 2SD of the IBDneg and pHC controls. Paired Wilcoxon test. Paired samples, *n* = 50. (B) LBP concentrations at baseline and visit 2 in UC patients with different types of induction therapy. Paired Wilcoxon tests. Corticosteroids (IV *n* = 5; oral *n* = 10), 5-ASA (oral + rectal *n* = 14; oral *n* = 18). (C) LBP concentrations in CD patients at time of diagnosis (baseline) and at visit 2 (after ∼3 months of induction therapy). Dashed line indicates the mean + 2SD of the IBDneg and pHC controls. Paired Wilcoxon test. *n* = 92 CD patients. (D) LBP concentrations at baseline and visit 2 in CD patients with different types of induction therapy. Paired Wilcoxon tests. Anti-TNF (infliximab *n* = 38; adalimumab *n* = 2), corticosteroids (CIR budesonide *n* = 7; oral *n* = 19), EEN (*n* = 22). (E) Correlation heatmap depicting correlations (*R*) of LBP with routinely used biomarkers at baseline (therapy-naive) and visit 2 (approximately 3 months after the start of induction therapy). Spearman correlations. (F) Correlation heatmap depicting correlations (*R*) of LBP and routinely used biomarkers with clinical activity scores (PUCAI in UC; wPCDAI in CD) at baseline (therapy-naive) and visit 2 (approximately 3 months after the start of induction therapy). Spearman correlations. *P < .05, **P < .01, ***P < .001, ****P < .0001.

To assess whether all therapy types are equally effective in reducing plasma LBP concentrations, we determined changes in longitudinal samples of patients who received different types of induction therapy (Figure 5B, D). In UC patients, corticosteroid induction therapy was associated with reduced LBP concentrations (Figures 5B and S5A, B). UC patients receiving 5-ASA induction therapy already had low plasma LBP at time of diagnosis, and as such, no effect of 5-ASA treatment on LBP concentrations could be observed (Figures 5B and S5A, B). In CD, plasma LBP concentrations at time of diagnosis were similar across patients who received different types of induction therapy (Figure S5C). In CD patients who received upfront anti-TNF or exclusive enteral nutrition (EEN) as induction therapy, plasma LBP concentrations were significantly reduced during and after the induction therapy period, while this was not observed in corticosteroid-treated CD patients (Figures 5D and S4D). This agrees with previous observations in the TISKIDS RCT, which demonstrated that 10 weeks of corticosteroid induction therapy is less effective in inducing endoscopic remission in CD.^39^^,^^60^ Hence, these data indicate that not all types of induction therapy result in a decrease in plasma LBP concentration, likely reflecting differences in successful remission induction.

### 3.7. After 12 weeks of induction therapy, changes in plasma LBP are associated with immune response dynamics in UC and CD

To investigate whether plasma LBP concentrations could aid in establishing whether remission has been achieved, we determined how plasma LBP relates to other biochemical plasma parameters and FCP after induction therapy, and how these parameters relate to clinical disease activity at this timepoint. After induction therapy in UC, LBP remained strongly correlated to CRP, but was less strongly correlated to other routinely used biomarkers compared to time of diagnosis (Figures 5E and S6A). In CD patients, LBP was generally more strongly correlated to other routinely used blood-based biomarkers after induction therapy compared to the therapy-naive time-point (Figures 5E and S6A). In agreement, in CD, LBP correlated moderately to FCP after the induction therapy phase (*R* = 0.43, *P* < .001), while this association was not present at time of diagnosis. This could be explained by the reduction in FCP concentration, leading to a more linear relationship with intestinal inflammation and barrier breach. In contrast to LBP at time of diagnosis, LBP concentrations after the induction therapy phase did not correlate with clinical disease activity scores in both UC and CD (Figures 5F and S6B). Similarly, the majority of other routinely used biomarkers were less strongly related to clinical disease activity after induction therapy compared to time of diagnosis. Clinical disease activity scores have previously been demonstrated to have more limited correlation with intestinal inflammation,^18^^,^^61^ which may explain the lack of relationship between clinical disease activity scores and fecal and blood-based biomarkers, including LBP. As such, elevated LBP concentrations in patients who are in clinical remission could be indicative of ongoing subclinical intestinal inflammation and barrier permeability.

As LBP at time of diagnosis was associated with immune responses in both UC and CD, we asked whether the reduction in LBP upon induction therapy was also associated with changes in the immune response. Indeed, the log_2_ fold change (FC) in LBP concentrations (from diagnosis to after approximately 12 weeks of induction therapy) was significantly correlated to the log_2_ FC of the majority of the immune proteins that were associated with LBP at the therapy-naive stage (Figure S6C). These results indicate that LBP does not only relate to immune responses at time of diagnosis, but also associates with changes in inflammatory immune responses that are associated with severity of UC and CD over time.^8^^,^^10^

### 3.8. Plasma LBP relates to clinical and endoscopic disease activity during disease course in both UC and CD

In view of the strong relationship of plasma LBP with clinical, endoscopic, histological, and immunological disease at diagnosis in IBD, particularly in UC, we hypothesized that plasma LBP may be a valuable parameter to assess dynamics of intestinal disease activity longitudinally. We therefore first assessed whether patients with high plasma LBP concentrations at diagnosis retain a high LBP concentration over time. Despite the strong reduction in LBP concentrations after therapy, UC and CD patients who were in the top quartile of LBP concentrations at time of diagnosis remained high in LBP concentrations during the first year after diagnosis (Figure 6A).

**Figure 6.**
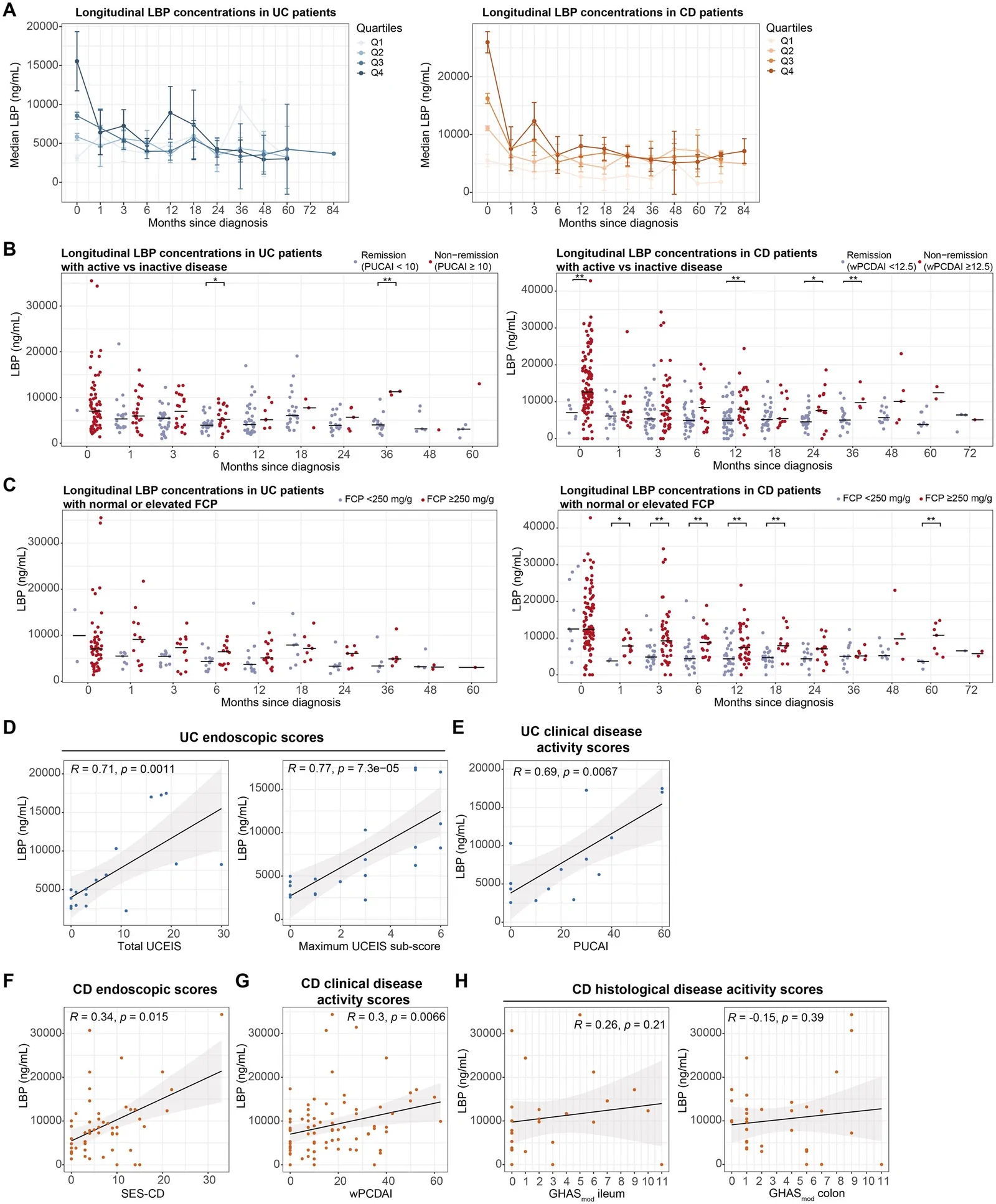
LBP associates with clinical and endoscopic disease activity during the disease course. (A) UC and CD patients were divided in quartiles based on their plasma LBP concentration at diagnosis. Each dot represents the median LBP concentrations per visit for each quartile. Error bars depict 95% confidence interval. (B) LBP concentrations in UC (left) and CD (right) patients at different follow-up visits, split by disease activity status (ie, remission [blue] or non-remission [red]). In UC, remission was defined as a PUCAI <10. In CD, remission was defined as a wPCDAI <12.5. Wilcoxon tests. (C) LBP concentrations in UC (left) and CD (right) patients at different follow-up visits, split out by normal (<250 µg/g; blue) vs elevated (≥250 µg/g; red) FCP. Wilcoxon tests. (D, E) Total UCEIS and maximum UCEIS sub-score (D), and PUCAI scores (E) in UC patients at follow-up endoscopy timepoints. (F–H) SES-CD (F), wPCDAI scores (G), and histological GHAS scores (H) in CD patients at follow-up endoscopy timepoints. Spearman correlations. *P < .05, **P < .01.

Next, we studied the relationship between LBP and clinical disease activity scores over time. We performed linear mixed models including LBP and time since diagnosis as fixed-effect predictors and accounting for repeated measurements within patients as a random effect. All visits, including unscheduled visits, were included in this model. In both UC and CD, higher LBP concentrations were associated with increased clinical disease activity over time. For each 1 µg/mL increase in LBP, the PUCAI score increased by 1.86 points in UC (Type II Wald χ^2^ = 71.4, *P* < .001) and the wPCDAI increased by 1.76 points in CD (χ^2^ = 206.8, *P* < .001). As expected, time since diagnosis was independently associated with lower disease activity in UC (β = −0.10 per week, χ^2^ = 37.3, *P* < .001) and CD (β = −0.078 per week, χ^2^ = 40.3, *P* < .001). These results indicate that during disease course LBP remains related to clinical disease activity in both UC and CD. To visualize this relationship between plasma LBP and disease activity during follow-up, we compared patients with no/mild disease activity (in UC: PUCAI <10; in CD: wPCDAI <12.5) versus active disease and patients with normal versus elevated FCP at standard follow-up hospital visits. In both UC and CD, LBP was increased during active disease compared to no/mild disease at several visits (Figure 6B). Since subclinical bowel inflammation can occur despite low clinical disease activity scores, we also assessed the relationship with FCP, which has previously been suggested to predict relapse in asymptomatic patients.^62^^,^^63^ In both UC and CD, LBP was increased when patients had elevated FCP, but this only reached statistical significance in CD (Figure 6C).

To further investigate whether high LBP may reflect (subclinical) intestinal inflammation during follow-up, we assessed how LBP associates with endoscopic and histological disease activity during the disease course. To this end, we collected the clinical, endoscopic, and histological disease activity scores obtained during follow-up endoscopies. These endoscopies were performed for a range of reasons, including suspicion of disease flare, monitoring after surgery, and as part of an RCT study protocol (only applicable to CD patients, TISKIDS^39^). In both UC and CD, we observed statistically significant correlations of plasma LBP with endoscopic disease activity scores (Figure 6D, F) and clinical disease activity scores (Figure 6E, G). The association between plasma LBP and endoscopic severity was again much stronger in UC (Figure 6D; total UCEIS *R* = 0.71; highest UCEIS sub-score *R* = 0.77) compared to CD (Figure 6F; SES-CD *R* = 0.34). Unfortunately, histological disease activity scores were only available for follow-up endoscopies of CD, and not UC, patients. As expected, in CD patients, LBP did not associate with histological disease activity (Figure 6H), in line with our observations for diagnostic endoscopies (see Figure 2). Together, these results demonstrate that LBP remains strongly associated with endoscopic and clinical disease activity during the disease course in UC patients, while in CD this association was less pronounced, suggesting that LBP may reflect (subclinical) intestinal inflammation during follow-up in UC.

## 4. Discussion

Here we establish in a well-characterized real-world cohort of therapy-naive patients with extensive longitudinal follow-up that plasma LBP, a surrogate marker of systemic exposure to endotoxins and a proxy for intestinal permeability, relates to clinical and endoscopic disease activity in both pediatric UC and CD patients at time of diagnosis and during disease course. However, agreeing with differences in disease pathogenesis between UC and CD, LBP responses are more linearly related to disease extent and intensity of histological damage in UC than in CD. Crucially, in UC at diagnosis, LBP was more strongly and independently associated with clinical, endoscopic, and histological disease activity compared to routinely used blood-based biomarkers and acute phase inflammatory mediators, including CRP and IL-6. Moreover, in UC, LBP provided additional information beyond the routinely used blood-based biomarkers in these statistical models for estimating clinicopathological scores. Intestinal permeability and consecutive translocation of bacteria and/or bacterial products may fuel the pathogenic immune response in IBD, causing a vicious cycle of intestinal damage. In line with this, LBP was associated with an IL-17-neutrophil-IFN-γ-related immune profile in UC and with an IFN-γ-related immune profile in CD; both of these immune signatures are associated with a severe disease course in the respective pediatric diseases.^9^^,^^10^ Plasma LBP concentrations may be a valuable parameter to monitor disease activity longitudinally, as upon induction therapy, LBP concentrations decreased in both UC and CD patients. Not all types of induction therapy were associated with a reduction in plasma LBP, possibly reflecting insufficient induction of (deep) remission and mucosal healing. Lastly, we demonstrate that LBP concentrations are associated with clinical and endoscopic disease activity during the disease course in both UC and CD, but again more strongly in UC, underscoring its putative value as a parameter for monitoring UC intestinal disease activity.

Our key finding is that thorough systematic analyses in a real-world pediatric IBD cohort uncover the value of the simple, well-known peripheral blood parameter LBP in capturing endoscopic disease severity and extent, intensity of histological disease activity, as well as degree of IL-17^+^ and calprotectin^+^ cell infiltration within a tissue biopsy in UC. The relationship between LBP and intestinal disease activity was stronger than—and independent from—other routinely used blood-based parameters, including CRP, ESR, albumin, and Hb at time of diagnosis. Moreover, LBP provided additive explanatory information beyond those routinely used blood-based biomarkers in the multivariable linear regression models and improved the models’ estimation of clinicopathological scores. The association between LBP and clinicopathological scores was maintained throughout disease course, thereby differentiating remission from flare. As such, measuring LBP could support clinical decision-making, in particular when invasive endoscopy is considered to assess a suspected flare. Implementation of LBP in clinical decision-making would require validation in a new pediatric cohort. As there are differences in the disease course of pediatric-onset compared with adult-onset IBD,^1^ additional validation in large, adult-onset IBD cohorts is needed. Already, preliminary evidence from a large adult IBD cohort supports our finding that plasma LBP relates to clinical and endoscopic disease activity (ECCO abstract^64^).

Intriguingly, despite the fact that LBP concentrations overall reached higher values in CD than in UC, LBP related differently to clinicopathological and immunological disease activity in these two disease entities. It could be questioned whether high LBP in isolated colonic CD would resemble UC in its association to clinicopathological features. As this cohort only contains 13 CD patients with isolated colonic disease, this could not be established. In CD, LBP significantly related to clinical disease activity, but had a weaker significant relationship with endoscopic severity (diagnosis, *R* = 0.28) than in UC (diagnosis, *R* = 0.71), did not significantly relate to histological disease activity in biopsies, and did not associate with disease location or granuloma. This implies that disease extent and intensity of immune infiltration may not be the main drivers of the high increases in plasma LBP in CD, and raises the question of which pathogenic process could account for the increase in LBP instead. A possible contributor to high plasma LBP in CD could be the transmural nature of the disease. Deep lesions in CD may lead to extensive translocation of bacteria and bacterial products and consequently high LBP, while only a small surface area of the intestine is affected. If this is the case, the strong reduction in plasma LBP concentrations observed in CD patients upon treatment initiation could be reflecting transmural healing, which is insufficiently captured by endoscopic and histological scoring. Future studies should investigate the relationship between LBP and the depth and number of transmural lesions in CD, for instance by using magnetic resonance enterography or intestinal ultrasound.

A unique aspect of our study is the extensive immune characterization next to detailed clinicopathological characterization of our real-world pediatric IBD cohort. Previously, we have identified specific disease “immunotypes” within pediatric UC and CD at time of diagnosis that strongly relate to development of a severe disease course.^8–10^ Here, we interrogated the relationship between plasma LBP and the immune response in each UC and CD patient. Theoretically, increased intestinal permeability could be a driver of increased immune activation in UC and CD, as bacterial or bacterial product translocation may fuel the chronic immune activation. The results of our plasma proteomic analyses, so far, favor an adjuvant-like mechanism, as increased LBP was associated with disease-specific immunotypes associated with severe disease in UC and CD. In CD, LBP correlated with the characteristic IFN-γ-related immune profile known to confer severe disease.^9^^,^^10^ In UC, high LBP concentrations were related to a predominance of the IL-17/neutrophil/IFN-γ-related plasma protein profile and increased infiltration of IL-17A^+^ and calprotectin^+^ cells in the intestinal lesions, which is typical of a severe disease course in UC.^10^

We also observed strong correlations of LBP with CRP, IL-6, and CSF-1, all proteins involved in the acute-phase response and associated with sepsis,^65–67^ raising the question of whether LBP provides new information beyond that captured by other acute-phase proteins. Acute-phase proteins comprise a diverse group of plasma proteins with distinct kinetics, regulatory pathways, and immunological functions. Therefore, although all these proteins may increase during systemic inflammation, they provide complementary rather than redundant information. The complementary value of LBP is supported by the results from our multivariable statistical analyses that demonstrated that the association between LBP and clinicopathological parameters was independent of IL-6 and CRP. In addition, we found that LBP in our therapy-naive CD and UC samples related to procalcitonin, a biomarker that is preferentially induced by bacterial infections and endotoxin exposure and used in clinical diagnostics^68–70^ (data not shown). Together, these data suggest that elevated LBP reflects bacterial product exposure in addition to nonspecific inflammation and provides complementary information to other acute-phase proteins.

Alternative to intestinal permeability being a driver of the disease-specific immune response, it could merely be a consequence of the breadth of the patient’s intrinsic pathogenic immune response and the intensity of the subsequent epithelial barrier damage, which may, for example, occur in UC where autoantibodies against the epithelial integrin α_v_β_6_ are hypothesized to directly damage the epithelial barrier.^71^ It is pivotal to disentangle whether intestinal permeability is a mere result or a driver of the pathogenic immune response, as numerous therapeutic interventions are designed to reinforce epithelial barrier integrity next to modulating the pathogenic immune response. In this context, it is important to emphasize that approximately 3 months of induction treatment with EEN was almost equally effective as up-front anti-TNF therapy in restoring LBP plasma concentrations in CD, indicating that both modulation of luminal microbiota by EEN^72^ and targeted modulation of the immune system by anti-TNF therapy restore intestinal barrier integrity. Our real-world data cohort is not suitable to disentangle whether loss of epithelial barrier integrity is a cause or consequence of the pathogenic immune response. Controlled studies with specific interventions, such as microbial therapy or local microbial metabolite supplementation to reinforce intestinal barrier integrity in patients receiving one specific immune-modulatory regimen, would have such potential. Our study emphasizes that plasma LBP is a highly informative surrogate parameter of intestinal barrier function that should be considered for inclusion in such studies. In our analyses, plasma LBP concentrations did not differ between CD patients with ileal versus colonic disease location, demonstrating that macroscopic anatomical location of intestinal barrier dysfunction has little, if any, influence on plasma LBP concentrations. In addition, others have demonstrated a strong correlation between plasma LBP concentrations and the lactulose–mannitol permeability assay, but not fecal calprotectin, in individuals with obesity, arguing that measuring LBP allows us to distinguish intestinal barrier dysfunction from inflammation.^25^ However, optimally designed controlled intervention studies could incorporate multiple parameters for intestinal permeability in addition to LBP, such as zonulin, which relates to epithelial tight junction integrity, and I-FABP, which is released upon epithelial damage,^27^ and combine those measures with magnetic resonance enterography or intestinal ultrasound.

In conclusion, we are the first to report that plasma LBP concentrations are associated with clinical, endoscopic, histological, and immunological measures of disease activity in pediatric IBD patients both at diagnosis and during follow-up, with particularly strong associations in UC, where LBP also reflected the extent of affected intestinal tissue. Besides adding to the understanding of the role of intestinal permeability in UC and CD pathogenesis, our finding that LBP provides incremental and complementary explanatory value beyond routinely used blood-based biomarkers for assessing disease activity in UC warrants (re)consideration of the use of this simple, inexpensive parameter for monitoring mucosal inflammation in UC.

## Supplementary Material

jjag146_Supplementary_Data

## Data Availability

The data underlying this article are available in DataverseNL, at https://doi.org/10.34894/O6WUS7
