# Supplementary material for: Plasma lipopolysaccharide-binding protein (LBP), a proxy for intestinal permeability, relates to clinicopathological and immunological disease activity in inflammatory bowel disease patients at diagnosis and during disease course
Source: J Crohns Colitis. 2026 Oct 9;20(10):jjag146. doi: 10.1093/ecco-jcc/jjag146 (PMC13652030; doi:10.1093/ecco-jcc/jjag146)
Supplement: Supplementary file 1 [file jjag146_supplementary_data.pdf]

Supplemental Material

Supplemental Figures

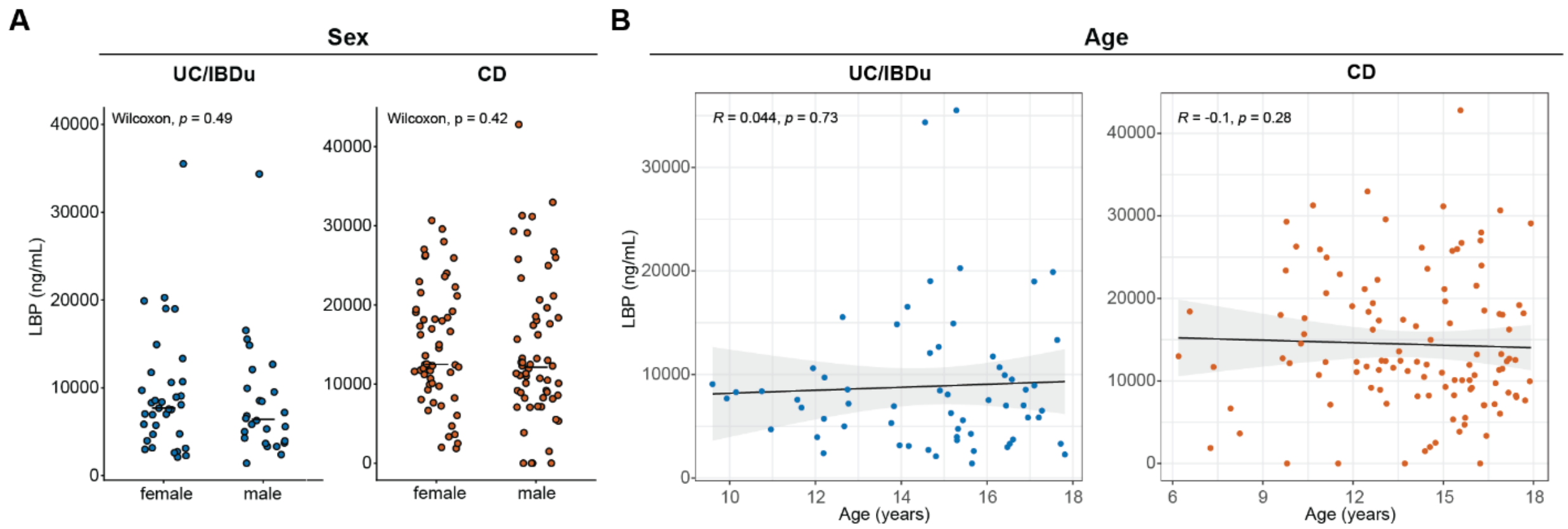

Figure S1. LBP does not associate with sex and age.

(**A**) LBP in female and male UC/IBDu and CD patients at time of diagnosis. Wilcoxon tests. (**B**) Correlation of LBP with age in UC/IBDu and CD patients at time of diagnosis.

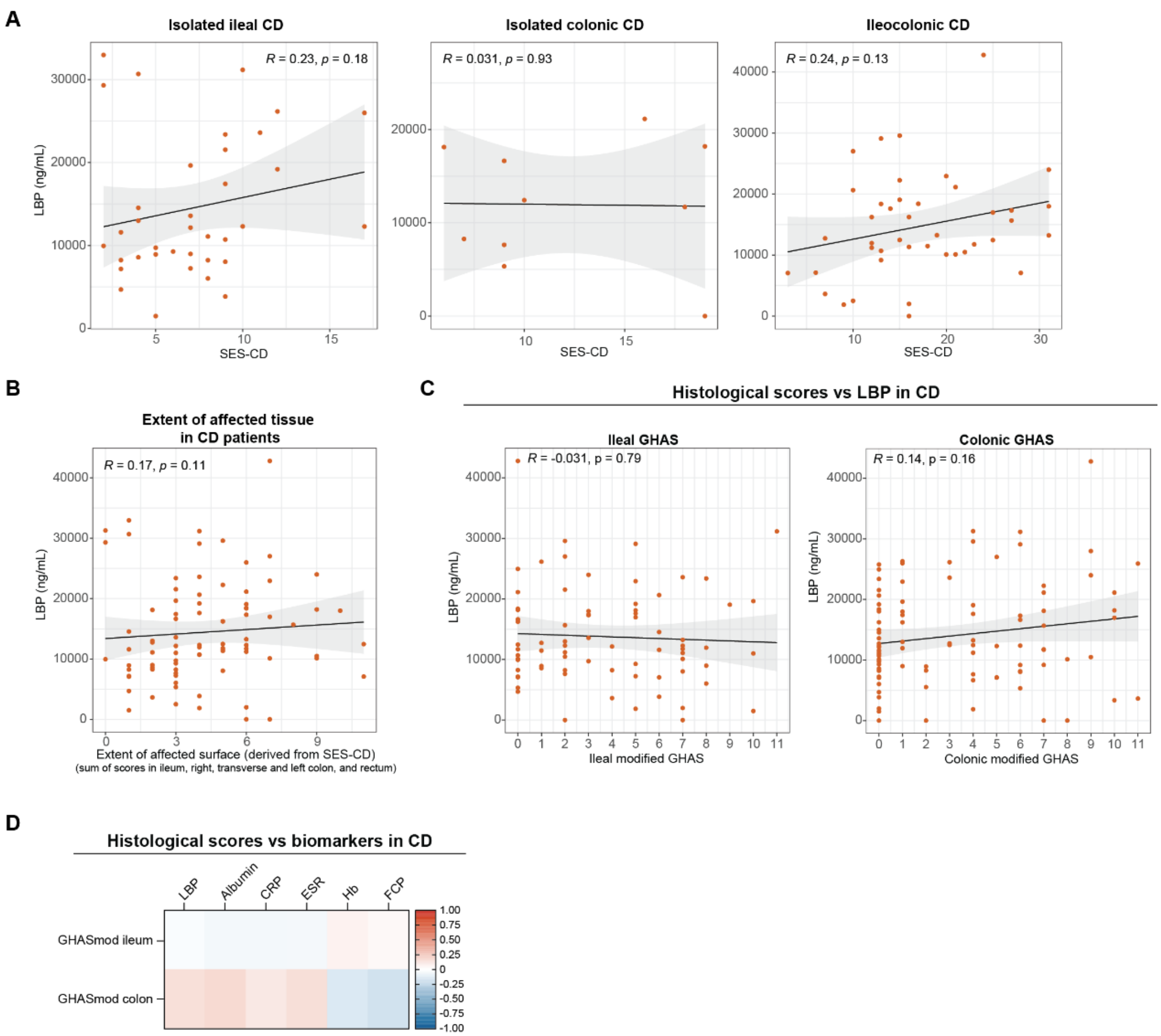

Figure S2. In CD, LBP does not relate to the extent of affected intestinal tissue and histological disease activity

Correlation of plasma LBP with (**A**) SES-CD in CD patients with isolated ileal (L1), isolated colonic (L2) and ileocolonic (L3) disease, (**B**) the sum of the "extent of affected surface" sub-score of the SES-CD in ileum, right, transverse and left colon, and rectum in CD patients and (**C**) with the ileal and colonic GHAS in CD patients (not selected for macroscopic ileal and colonic involvement) at time of diagnosis. Spearman's correlations. (**D**) Heatmap of Spearman's correlations of ileal and colonic GHAS scores in all CD patients (not selected for ileal or colonic involvement) with biomarkers at time of diagnosis. Color represents the *R*.

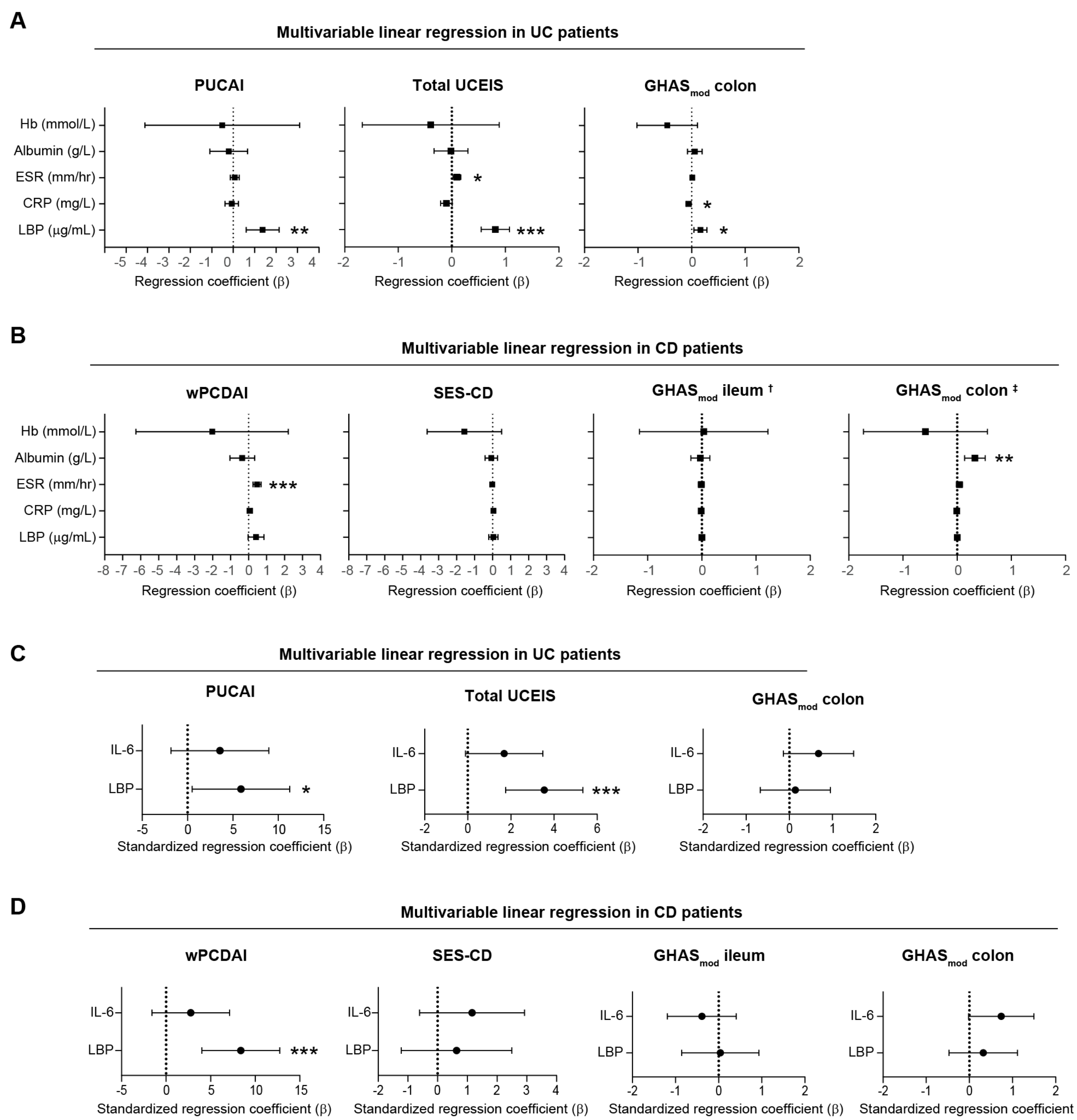

Figure S3. Plasma LBP is independently associated with disease scores in UC, but not in CD, at time of diagnosis.

Forest plots depicting the (standardized) regression coefficients (β) and 95% confidence intervals (CI) of multivariable linear regression models. Positive coefficients indicate a positive association with the outcome, whereas negative coefficients indicate an inverse association. (**A-B**) Models included LBP, CRP, ESR, albumin and Hb as predictor covariates and clinical, endoscopic and histological disease scores as dependent variables in UC (**A**) and CD (**B**). Covariates were not standardized. †: in selection of patients with macroscopic ileal involvement, ‡: in selection of

patients with macroscopic colonic involvement, based on the disease location according to the Paris classification. (**C-D**) Models included LBP and IL-6 as predictor covariates and clinical, endoscopic and histological disease scores as dependent variables in UC (**C**) and CD (**D**). Covariates were standardized. *$p<0.05$, **$p<0.01$, ***$p<0.001$.

A

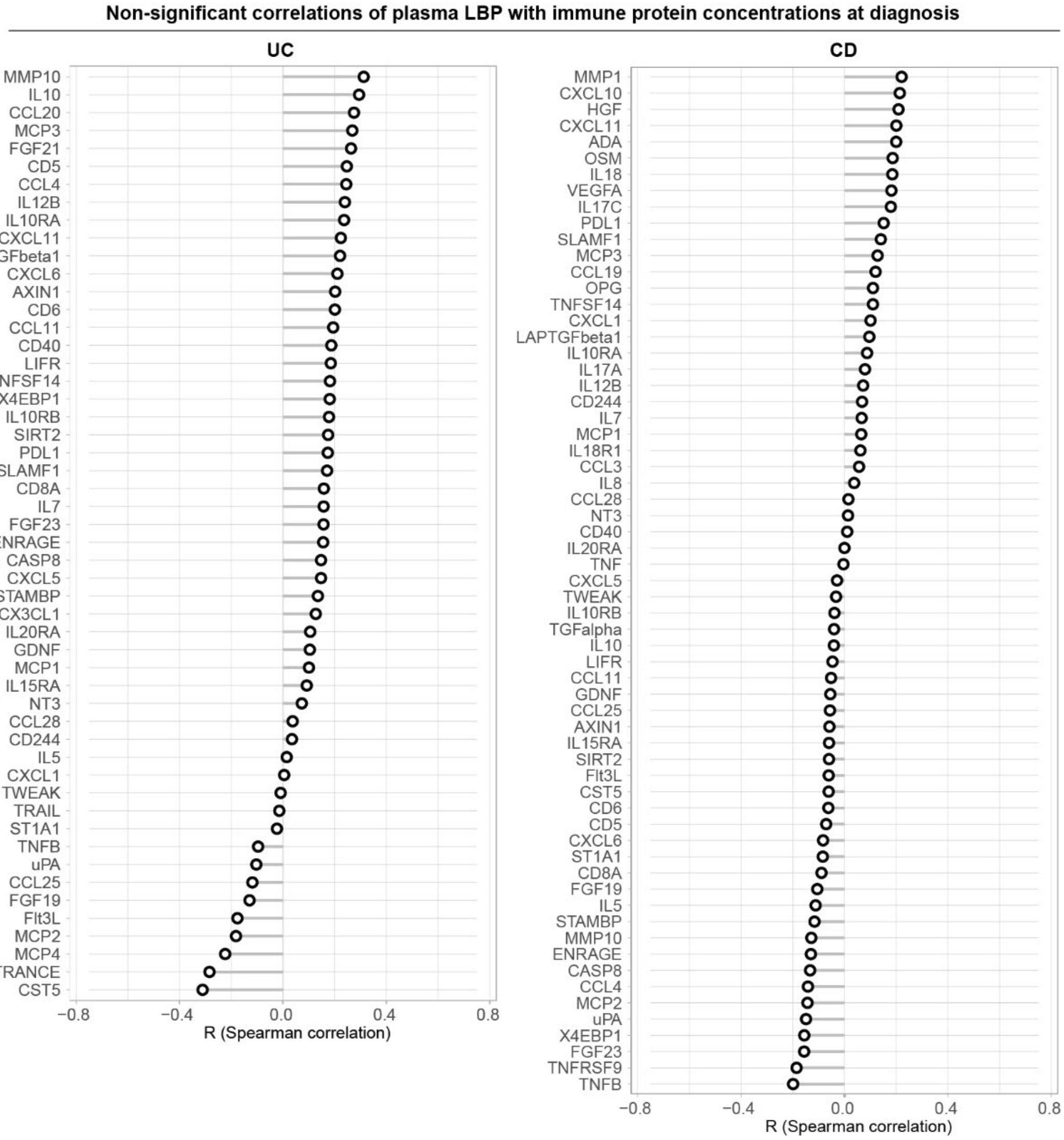

Figure S4. Non-significant correlations of LBP with plasma immune proteins at time of diagnosis in UC and CD. (**A**) Lollipop plots representing the non-significant correlations (R) between LBP and immune protein concentrations in plasma. Significant correlations can be found in Figure 4B. Spearman's correlations followed by Benjamin-Hochberg correction. Samples size: CD n=115 and UC n=58.

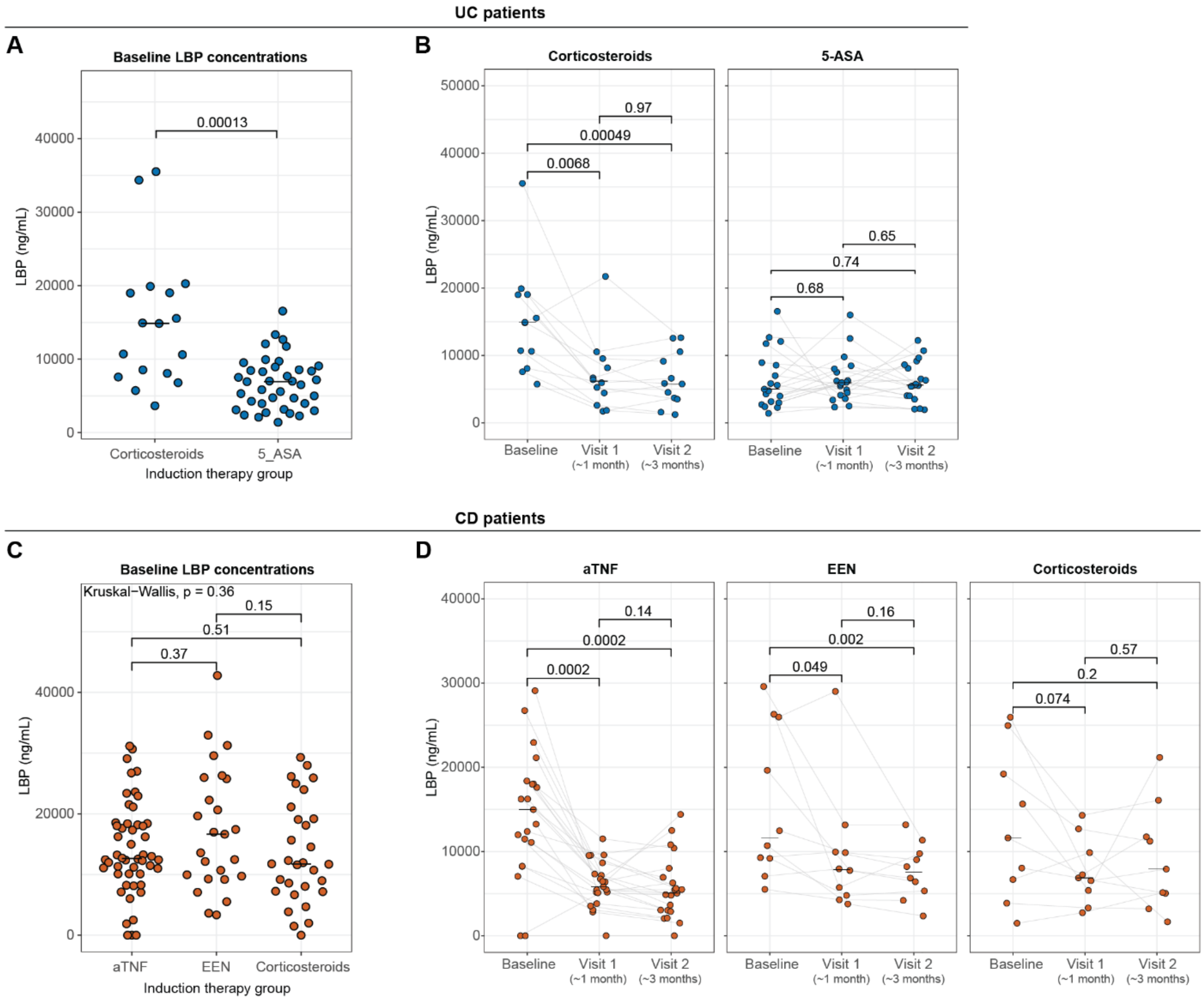

Figure S5. LBP concentrations are reduced during and after induction therapy by some but not all types of induction therapy

(**A**) LBP concentrations at diagnosis in UC patients that received different types of induction therapy. Wilcoxon test. (**B**) LBP concentrations at baseline, visit 1 and visit 2 in UC patients with different types of induction therapy. Paired Wilcoxon tests. Corticosteroids (IV n=4; oral n=8), 5-ASA (oral+rectal n=9; oral n=10). (**C**) LBP concentrations at baseline in CD patients that received different types of induction therapy. Kruskal-Wallis and Wilcoxon tests. (**D**) LBP concentrations at baseline, visit 1 and visit 2 in CD patients with different types of induction therapy. Paired Wilcoxon tests. Anti-TNF (infliximab n=21), EEN (n=10), corticosteroids (CIR budesonide n=3; oral n=6)).

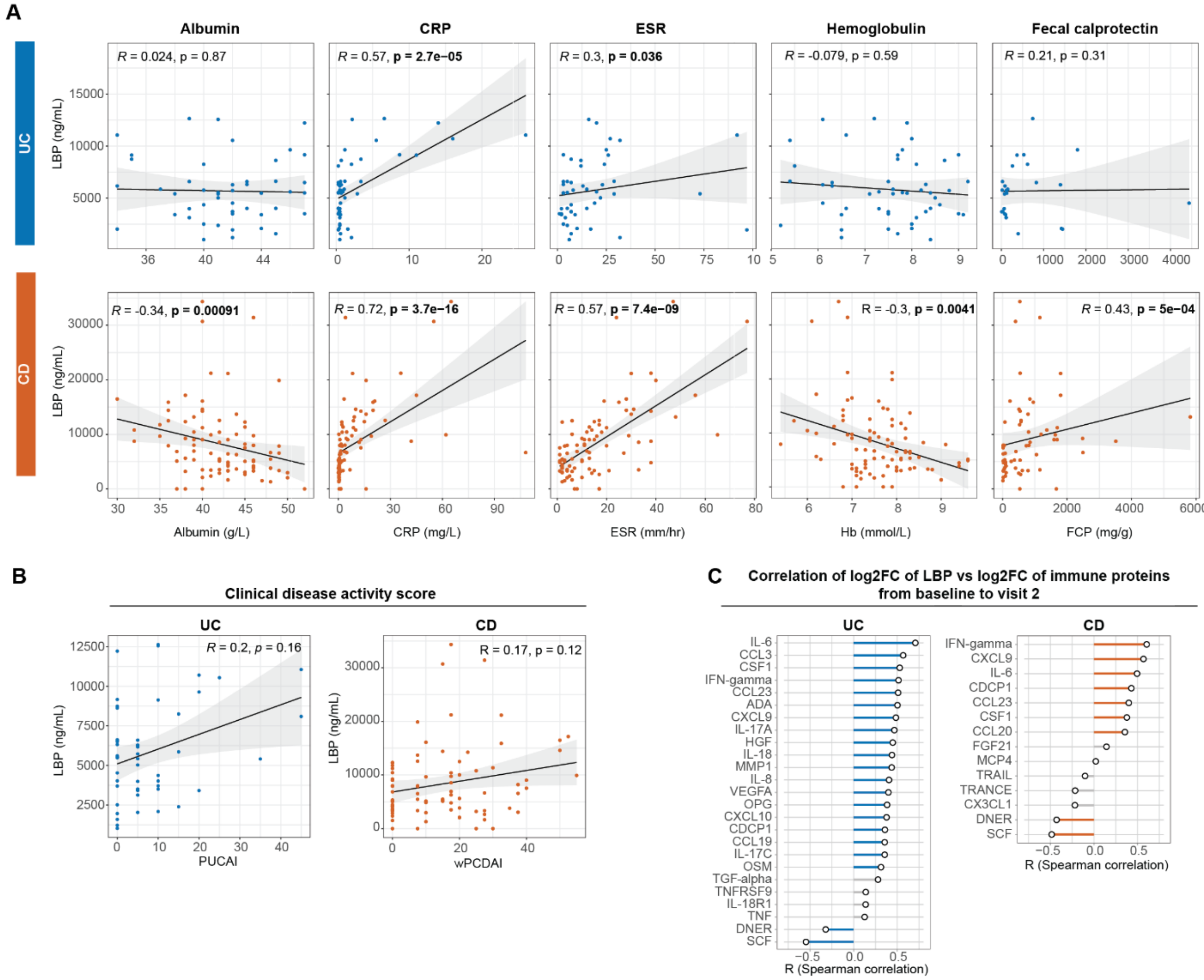

Figure S6. LBP associates with some blood-based biochemical parameters and fecal calprotectin, primarily in CD, but not with clinical disease activity after the induction therapy phase

(**A**) Correlations of LBP concentrations vs. albumin, CRP, ESR, hemoglobin (Hb) and fecal calprotectin (FCP) in UC (top) and CD (bottom) at visit 2 (after induction therapy). Spearman's correlations. (**B**) Correlations of LBP with clinical disease activity scores in UC (PUCAI) and CD (wPCDAI) at visit 2. (**C**) Lollipop graphs representing the correlation (R) between the log2FC of LBP and the log2FC of immune protein concentrations in plasma from baseline to visit 2 (approximately 3 months after the start of induction therapy). Only immune proteins found to be significantly correlated to LBP at baseline (see Figure 4) were included. Colored lines indicate p-adj < 0.05. Spearman correlations with Benjamin-Hochberg correction.

Supplemental Tables

Supplemental table 1. Timing of scheduled PIBD-SETQ study visits relative to therapy initiation

Visit number	n	Median time since start induction therapy (weeks)	IQR (25%-75% percentile)
1	91	5.1	4.000 - 6.143
2	123	13.0	11.57 - 15.00
3	113	26.3	24.36 - 29.14
4	108	52.0	49.71 - 54.86
5	89	78.0	74.14 - 80.07
6	75	103.9	100.3 - 106.4
7	53	155.0	152.3 - 158.1
8	31	206.9	204.1 - 209.3
9	23	261.0	259.1 - 266.0
10	11	307.7	303.7 - 312.7
11	4	366.7	362.2 - 369.9

Supplemental table 2: antibodies used for immunohistochemistry

Target protein	Serum	Primary antibody	Secondary antibody
IL-17A	Normal rabbit serum (Jackson ImmunoResearch, West Grove, PA, USA)	Anti-human IL-17/IL17A polyclonal goat IgG antibody, 2 ug/mL (R&D Systems/Bio-Techne, Minneapolis, MN, USA; AF-317)	Rabbit-anti-goat antibody, 1:500 (Vector Laboratories)
Calprotectin (S100A8/A9)	Normal horse serum (Biowest)	Anti-human S100A8/A9 heterodimer monoclonal mouse IgG1 antibody, 1.2 ug/mL (R&D Systems/BioTechne; MAB45702)	Horse-anti-mouse antibody, 1:500 (Vector Laboratories)
